# Comprehensive Proteome and Lysine Acetylome Analysis Reveals the Widespread Involvement of Acetylation in Cold Resistance of Pepper (*Capsicum annuum* L.)

**DOI:** 10.3389/fpls.2021.730489

**Published:** 2021-08-27

**Authors:** Zhoubin Liu, Jingshuang Song, Wu Miao, Bozhi Yang, Zhuqing Zhang, Wenchao Chen, Fangjun Tan, Huan Suo, Xiongze Dai, Xuexiao Zou, Lijun Ou

**Affiliations:** ^1^College of Horticulture, Hunan Agricultural University, Changsha, China; ^2^ERC for Germplasm Innovation and New Variety Breeding of Horticultural Crops, Changsha, China; ^3^Key Laboratory for Vegetable Biology of Hunan Province, Changsha, China; ^4^Vegetable Research Institute, Hunan Academy of Agricultural Science, Changsha, China; ^5^Hunan Xiangyan Seed Industry Co., Ltd, Changsha, China

**Keywords:** acetylation modification, cold stress, carbon fixation, pepper, proteome, photosynthesis, recovery

## Abstract

Pepper is a typical warmth-loving vegetable that lacks a cold acclimation mechanism and is sensitive to cold stress. Lysine acetylation plays an important role in diverse cellular processes, but limited knowledge is available regarding acetylation modifications in the resistance of pepper plants to cold stress. In this study, the proteome and acetylome of two pepper varieties with different levels of cold resistance were investigated by subjecting them to cold treatments of varying durations followed by recovery periods. In total, 6,213 proteins and 4,574 lysine acetylation sites were identified, and this resulted in the discovery of 3,008 differentially expressed proteins and 768 differentially expressed acetylated proteins. A total of 1,988 proteins were identified in both the proteome and acetylome, and the functional differences in these co-identified proteins were elucidated through GO enrichment. KEGG analysis showed that 397 identified acetylated proteins were involved in 93 different metabolic pathways. The dynamic changes in the acetylated proteins in photosynthesis and the “carbon fixation in the photosynthetic organisms” pathway in pepper under low-temperature stress were further analyzed. It was found that acetylation of the PsbO and PsbR proteins in photosystem II and the PsaN protein in photosystem I could regulate the response of pepper leaves to cold stress. The acetylation levels of key carbon assimilation enzymes, such as ribulose bisphosphate carboxylase, fructose-1,6-bisphosphatase, sedoheptulose-1,7-bisphosphatase, glyceraldehyde 3-phosphate dehydrogenase, phosphoribulokinase, and triosephosphate isomerase decreased, leading to decreases in carbon assimilation capacity and photosynthetic efficiency, reducing the cold tolerance of pepper leaves. This study is the first to identify the acetylome in pepper, and it greatly expands the catalog of lysine acetylation substrates and sites in Solanaceae crops, providing new insights for posttranslational modification studies.

## Introduction

Pepper (*Capsicum annuum* L.) is one of the most important vegetable crops in the world, and it is regarded as being a good source of basic nutrients, including capsaicinoids, pigments (anthocyanins and carotenoids), and vitamins (Aza-González et al., 2011). It is a typical warmth-loving vegetable that lacks a cold acclimation mechanism and is sensitive to cold stress; the optimal temperature range for its growth is 21–28°C. Cold stress is a major abiotic stress factor, and it can cause physiological damage in plants, including blocking the synthesis of chlorophyll and inhibiting photosynthetic enzymes (Aroca et al., 2003). A previous study found that cold stress can lead to a decrease in the number of pollen grains and an imbalance in antioxidant metabolism and homeostasis (Airaki et al., 2012). Cold stress has thus become the main factor restricting the growth and development of pepper plants.

In recent years, transcriptome tools have become useful for studying the molecular regulation mechanisms of cold adaptation in plants, and a large number of genes involved in the regulation of cold tolerance have been identified (Liu et al., 2012; Yang et al., 2015). Proteomic techniques have also been used to further study the mechanisms of plant cold tolerance. For example, Zhang et al. (2016) quantified 117 differentially expressed proteins (DEPs) under cold stress in petunia seedlings, and most of these proteins were involved in oxidation–reduction processes. Using two-dimensional gel electrophoresis, Gharechahi et al. (2014) identified 34 proteins that changed significantly in the response of wild wheat to cold stress, including several cold-stress-related proteins, cold-regulated proteins, cold-responsive LEA/RAB-related COR proteins, and oxygen-evolving enhancer proteins. Using two-dimensional differential in-gel electrophoresis, Kosová et al. (2013) identified 36 DEPs in spring and winter wheat under cold stress; they found an increased abundance of proteins involved in carbohydrate catabolism, redox metabolism, and chaperones, as well as defense-related proteins.

Precursor proteins need to undergo a series of posttranslational modifications (PTMs) to form functionally active proteins. It is widely accepted that protein PTM of targets is a critical process in transmitting environmental stresses to physiologically diverse processes, including cell cycle regulation, growth, enzyme activation, and protein stability (Philp et al., 2014). *N*^ε^-lysine acetylation is a reversible PTM that can control enzyme activities and carbon flux through different metabolic pathways and hormonal signaling. It plays key roles in a number of processes, including the cell cycle, flowering time, responses to environmental conditions, such as light or pathogen attack, root and shoot development, hormone signaling, and epigenetic processes (Xing and Poirier, 2012; Hart and Ball, 2013). Histone and many other non-histone proteins have been found to be acetylated, and their roles in regulating gene transcription have been extensively investigated (Li et al., 2007). Overexpression of 35S:*AtHD2C-GFP* in transgenic *Arabidopsis* resulted in reduced transpiration and enhanced tolerance to salt and drought stresses (Sridha and Wu, 2006). *AtHDA6* expression in *Arabidopsis thaliana* was upregulated by cold (2°C) stress, and the *HDA6* mutant *axe1-5* was highly sensitive to low temperatures (−18°C) after low-temperature acclimation (To et al., 2011). Hu et al. (2011) reported that cold treatment induces significant upregulation of histone deacetylases (HDACs), leading to global deacetylation of histones H3 and H4, and trichostatin A treatment could inhibit the induction of cold-response genes such as *ZmDREB1* and *ZmCOR413*, indicating that HDAC activity is necessary for cold-stress response and can activate the transcription of cold-induction genes. Recently, several large-scale lysine acetylome studies have been performed in species, including *A. thaliana* (Finkemeier et al., 2011), soybean (Smith-Hammond et al., 2014), and strawberry (Fang et al., 2015). Unfortunately, to date, there have been no studies focusing on large-scale screening of protein acetylation under cold stress in plants.

There have been reports on the effects of cold stress on the photoinhibition characteristics and photosynthetic rate of pepper, but the research has been focused on the effects of cold stress on physiological and biochemical characteristics (Pivonia et al., 2012; Ou et al., 2015). Therefore, to better understand the differences in the cold-tolerance adaptation mechanisms of peppers in the cold-stress and recovery stages, we present here for the first time comprehensive proteome and acetylation proteome profiling of two pepper varieties with different levels of cold resistance. Our findings provide important information on the molecular basis of cold resistance adaptation in this species, and this may be useful for further improving its cold resistance.

## Materials and Methods

### Plant Growth and Sampling

Two pepper (*Capsicum annuum* L.) varieties, cold-resistant A188 and cold-sensitive A122, were provided by Hunan Vegetable Research Institute (Changsha, Hunan Province, China). Seeds were sown in 10 cm × 10 cm plastic pots with the nutrient substrate (organic matter ≥ 20%, N + P_2_O_5_ + K_2_O: 1–5%, pH: 5–7) and grown in a greenhouse (16 h of light at 30 ± 2°C and 8 h of darkness at 20 ± 2°C). After growing to four leaves and one core stage, the seedlings were treated in an artificial climate chamber (An extra compressor was added) (Cu-41L4, Percival Scientific Inc., United States) with temperature of 4°C, light intensity of 200 μmol/m2/s, air humidity of 70% and photoperiod of 16 h/8 h for different times up to 72 h, and then put at room temperature for recovery. In the whole treatment of cold stress and recovery, 10 experimental conditions, including 0, 2, 4, 8, 12, 24, 48, and 72 h of cold stress, recovery 1 h after 72-h cold stress, recovery 4 h after 72-h cold stress, were set. Each treatment was repeated three times, with a total of nine pepper seedlings per repetition (Supplementary Figure 1). After the cold stress time reached the preset time of each treatment, plant leaves were frozen in liquid nitrogen then stored at −80°C until analyses.

Subsequently, we applied 10 treatment samples to western blotting, using a pan anti-crotonyllysine antibody, a pan anti-2-hydroxyisobutyryllysine antibody, a pan anti-succinyllysine antibody, and a pan anti-acetyllysine antibody. It was found that acetylation modification of each treated protein was the most obvious PTM under cold stress (Supplementary Figure 2). Western blotting results were used to further select the four treatment samples, including 0 h of cold stress (T0), 2 h of cold stress (T1), 12 h of cold stress (T2), and recovery 1 h after 72 h of cold stress (R1) treatment for physiological measurement, proteome, and acetylome analysis.

### Measurements of Malonaldehyde, Total Soluble Sugar, Glutathione Reductase Activity, and Water Content

The content of total soluble sugar was determined using the anthrone method (Min et al., 2014). Malonaldehyde content was measured to evaluate the peroxidation of membrane lipids, using a previously established method (Lv et al., 2011). Glutathione reductase activity was determined using the previously established method (Ciacka et al., 2019).

Leaf water content is calculated by referring to the following formula:

$$\mathrm{Water}\mathrm{content}=\frac{{\text{W}}_{\text{f}}-W_{\text{d}}}{W_{\text{f}}}\times 100\%$$

W represents weight, Wf represents fresh weight, and Wd represents dry weight.

### Transmission Electron Microscope and Observation of Pepper Leaves

The samples were fixed in 3% pentadiol for 2 h, followed by two rounds of 10-min rinse with 0.1 mol/L phosphate buffer solution, and then soaked in 1% osmium acid for 5 h, followed by the same cleaning process. Acetone gradient dehydration and two rounds of pure acetone dehydration were then applied in the cleaned samples. Then, gradient osmotic was conducted, using 812 resins in a gradient rate of 3:1, 3:2, and 3:3. The samples were then placed in dry environment for 8 h with the container cap opened. Afterward, the samples were embedded, and then treated with gradient polymerization, sectioned, stained, and observed under a transmission electron microscope.

### Proteome Analysis

#### Protein Extraction

Samples were first grinded by liquid nitrogen, and then the powder was transferred to a 5-ml centrifuge tube and sonicated three times on ice, using a high intensity ultrasonic processor (Scientz, Ningbo, China) in a lysis buffer (including 1% Protease Inhibitor Cocktail, 3-μM Trichostatin A and 50-mM Nicotinamide). An equal volume of Tris-saturated phenol (pH 8.) was added; then, the mixture was further vortexed for 5 min. After centrifugation (4°C, 10 min, 5,500 *g*), the upper phenol phase was transferred to a new centrifuge tube. Proteins were precipitated by adding five volumes of ammonium sulfate-saturated methanol and incubated at −20°C for at least 8 h. After centrifugation at 4°C for 10 min, the supernatant was discarded. The remaining precipitate was washed with ice-cold methanol once, followed by ice-cold acetone for three times. The protein was redissolved in 8-M urea, and the protein concentration was determined with a BCA kit (Beyotime Biotechnology, Shanghai, China) according to the instructions of the manufacturer.

#### Trypsin Digestion

For digestion, the protein solution was reduced with 5-mM dithiothreitol for 30 min at 56°C and alkylated with 11-mM iodoacetamide for 15 min at room temperature in darkness. The protein sample was then diluted by adding 100-mM Triethylammonium bicarbonate to urea concentration less than 2 M. Finally, trypsin was added at a 1:50 trypsin-to-protein mass ratio for the first digestion overnight and a 1:100 trypsin-to-protein mass ratio for a second 4 h-digestion.

#### LC-MS/MS Analysis

The tryptic peptides were dissolved in 0.1% formic acid (solvent A), directly loaded onto a 25 cm × 75 μm analytical column, 1.6-μm C18 beads with an integrated nanospray emitter (IonOpticks, Australia). The gradient was composed of an increase from 6 to 24% solvent B (0.1% formic acid in acetonitrile) over 70 min, 24–32% in 14 min, and climbing to 80% in 3 min, and then holding at 80% for the last 3 min, all at a constant flow rate of 300 nl/min on a nanoElute UHPLC system (Bruker Daltonics Inc, Billerica, United States).

The peptides were subjected to Capillary source, followed by the timsTOF Pro (Bruker Daltonics Inc, Billerica, United States) mass spectrometry. The electrospray voltage applied was 1.4 kV. Precursors, and fragments were analyzed at the TOF detector, with an MS/MS scan range from 100 to 1,700 m/z. The timsTOF Pro was operated in a parallel accumulation serial fragmentation (PASEF) mode. Precursors with charge states 0 to 5 were selected for fragmentation, and 10 PASEF-MS/MS scans were acquired per cycle. The dynamic exclusion was set to 30 s.

#### Database Search

The resulting MS/MS data were processed, using Maxquant search engine (v.1.6.6.0), and LFQ (Label Free Quantitation) was used for quantitative analysis. The search was performed, using the following settings based on the pepper transcriptome database (unpublished) concatenated with a reverse decoy database. Trypsin/P was specified as a cleavage enzyme, allowing up to two missing cleavages. The mass tolerance for precursor ions was set as 40 ppm in the first search and the main search, and the mass tolerance for fragment ions was set as 40 ppm. Carbamidomethyl on Cys was specified as fixed modification, methionine oxidation, protein N-terminal acetylation, and deamidation (NQ) as variable modifications. FDR was adjusted to <1%, and a minimum score for modified peptides was set >40. Proteins identified in at least two of the three replicates in at least one sample were considered for expression analysis. For LFQ, while *t*-test *p* < 0.05, a fold change of >1.5 or <0.667 was regarded as significantly up- or downregulated, respectively.

#### Gene Ontology (GO) Annotation

Sequences of identified proteins were retrieved from the UniProt-GOA database^1^. The retrieved sequences were locally searched against the non-redundant protein sequence (NR) database, using the NCBI BLAST+ and InterProScan (v.5.14-53.0^2^) to find homologous sequences from which the functional annotation can be transferred to the studied sequences. Blast2GO was then used to annotate sequences and map gene ontology (GO) (version 3.3.5). The GO annotation results were plotted by R scripts.

#### KEGG Pathway Annotation

The sequences of identified proteins were blasted against the online Kyoto Encyclopedia of Genes and Genomes (KEGG) database^3^ to retrieve their KOs and were subsequently mapped to pathways in KEGG. The corresponding KEGG pathways were extracted.

#### Functional Enrichment Analysis

To further explore the impact of differentially expressed proteins on different biological processes and discover internal relations between differentially expressed proteins, enrichment analysis was performed. GO term and KEGG pathway enrichment analyses were performed based on Fisher’s exact test, considering the whole quantified protein annotations as the background dataset. Only functional categories and pathways with *p* < 0.05 were considered as significant.

### Acetylation Protein Analysis

#### Acetylation Protein Analysis

The method of protein extraction and trypsin digestion is the same as that described in proteome analysis.

#### Pan Antibody-Based PTM Enrichment

To enrich modified peptides, tryptic peptides dissolved in a immunoprecipitation (IP) buffer (100-mM NaCl, 1 mM EDTA, 50-mM Tris-HCl, 0.5% NP-40, pH 8.0) were incubated with prewashed acetylated beads (Lot No. 104, PTM Bio) at 4°C overnight with gentle shaking. Then, the beads were washed four times with an IP buffer and two times with H_2_O. The bound peptides were eluted from the beads with 0.1% trifluoroacetic acid. Finally, the eluted fractions were combined and vacuum-dried. For LC-MS/MS analysis, the resulting peptides were desalted with C18 ZipTips (Millipore Inc., MA, United States) according to the instructions of the manufacturer.

#### LC-MS/MS Analysis

The tryptic peptides were dissolved in 0.1% formic acid (solvent A), directly loaded onto a 25 cm × 75 μm analytical column, 1.6-μm C18 beads with an integrated nanospray emitter (IonOpticks, Australia). The gradient was comprised of an increase from 6 to 22% solvent B (0.1% formic acid in acetonitrile) over 20 min, 22–30% in 6 min and climbing to 80% in 2 min and then holding at 80% for the last 2 min, all at a constant flow rate of 250 nl/min on a nanoElute UHPLC system (Bruker Daltonics Inc., Billerica, United States).

The peptides were subjected to the Capillary source, followed by the timsTOF Pro (Bruker Daltonics Inc., Billerica, United States) mass spectrometry. The electrospray voltage applied was 1.4 kV. Precursors, and fragments were analyzed at the TOF detector, with an MS/MS scan range from 100 to 1,700 m/z. The timsTOF Pro was operated in a parallel accumulation serial fragmentation (PASEF) mode. Precursors with charge states 0–5 were selected for fragmentation, and 10 PASEF-MS/MS scans were acquired per cycle. The dynamic exclusion was set to 24 s.

#### Database Search

The resulting MS/MS data were processed, using Maxquant search engine (v.1.6.6.0). Tandem mass spectra were searched against the pepper transcriptome database (unpublished) concatenated with a reverse decoy database. Trypsin/P was specified as a cleavage enzyme, allowing up to four missing cleavages. The mass tolerance for precursor ions was set as 40 ppm in the first search and the main search, and the mass tolerance for fragment ions was set as 40 ppm. Carbamidomethyl on Cys was specified as fixed modification, methionine oxidation, protein N-terminal acetylation, deamidation (NQ), and lysine acetylation as variable modifications. FDR was adjusted to <1%, and a minimum score for modified peptides was set >40. Proteins identified in at least two of the three replicates in at least one sample were considered for expression analysis. For modification sites intensity, while *t*-test *p* < 0.05, a fold change of >1.5 or <0.667 was regarded as significantly up- or downregulated, respectively.

#### GO, KEGG Annotation, and Enrichment Analysis

Gene Ontology, KEGG annotation, and enrichment analysis, used the same method described in proteome analysis.

#### Motif Analysis

Soft MoMo (motif-x algorithm) (V5.0.2^4^) was used to analyze the model of sequences constituted with amino acids in specific positions of modify-21-mers (10 amino acids upstream and downstream of the site) in all protein sequences. All the database protein sequences were used as a background database parameter. Minimum number of occurrences was set to 20. Emulate original motif-x was ticked and other parameters with default.

#### Subcellular Localization

WoLF PSORT (v.0.2^5^), a subcellular localization predication program, was used to predict subcellular localization of the proteins.

### Production of Polyclonal Antibodies, Immunoprecipitation, and Western Blotting Analysis

Anti-PsaN, anti-PsbO, anti-PsaN-K155acetyl, and anti-PsbR-K44acetyl polyclonal antibodies were generated by HUABIO Inc. (Hangzhou, Zhejiang, China). Immunoprecipitation using anti-PsaN and an anti-PsbO antibody, and western blotting analysis, using anti-PsaN, anti-PsbO, anti-PsaN-K155acetyl, and Anti-PsbR-K44acetyl antibodies, were performed as previously described (Mo et al., 2015).

## Results and Discussion

### Phenotype, Physiology, and Cell Microstructure Response of Peppers Under Cold Stress

**FIGURE 1:**
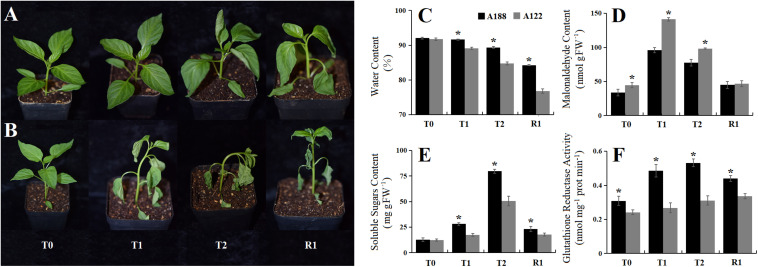
Phenotypic and physiological response of A188 (cold-resistant variety) and A122 (cold-sensitive variety) under cold stress and recovery stages. **(A)** Phenotype of cold-resistant A188 under different treatments. **(B)** Phenotype of cold-sensitive A122 under different treatments. **(C)** Changes in relative water content in the pepper leaves. **(D)** Changes in malondialdehyde content in the pepper leaves. **(E)** Changes in total soluble sugar contents in the pepper leaves. **(F)** Changes in glutathione reductase activity in the pepper leaves. T0, T1, T2, and R1 refer to 0 h, 2 h, 12 h of cold stress, and recovery 1 h after 72 h of cold stress treatment, respectively. The black and gray columns in the bar chart represent A188 and A122, respectively. The symbol * indicates a significant difference between A188 and A122 in the same treatment (*P* < 0.05).

Through phenotypic investigation, it was found that injury to A188 under cold stress appeared later and the plant recovered more easily, while the damage under cold stress to A122 appeared earlier, and it was difficult for the plant to recover (Figures 1A,B and Supplementary Figure 1). Malonaldehyde reflects oxidative injury of membranes; soluble sugars are important osmotic regulatory substances in plants, especially under cold stress; changes in relative water content probably reflect the degree of stress, whereas glutathione reductase activity is an enzyme involved in protection from oxidative stress, which usually accompanies cold stress. In this study, the application of cold stress caused these indexes to first increase and then decrease in both pepper varieties (Figures 1C–F). The leaf water content, soluble sugars, and glutathione reductase activity in A188 were significantly higher than those in A122, while the malonaldehyde content was significantly lower in A188 than in A122. The results of transmission electron microscopy showed that, with increasing cold-stress duration, the mesophyll cells gradually swelled, the cytoplasm separated from the cell wall, and the degree of damage to organelles deepened (Supplementary Figure 3). However, with the same cold-stress treatment, the degree of damage to A188 was lower than that to A122. Phenotypically and biochemically, A188 behaves as being cold tolerant, whereas A122 is a cold-sensitive variety.

### Cold Stress Changes the Proteome Profile in Pepper Seedlings

To examine the whole proteome in response to cold stress, four treatment stages were analyzed in seedlings of the two pepper varieties. The proteomic analysis identified 37,025 (91.34%) unique peptides from a total of 40,536 detected peptides (a false discovery rate, FDR ≤ 0.01). In total, 6,213 protein groups (FDR ≤ 0.01) were identified based on these peptides, among which 5,161 protein groups were quantified (Supplementary Table 1). Among these proteins, 4,220 of 5,006 and 4,058 of 5,976 quantified proteins were found in all four stages in A188 and A122, respectively (Supplementary Figure 4A).

### Statistics of DEPs Under Cold Stress

**FIGURE 2:**
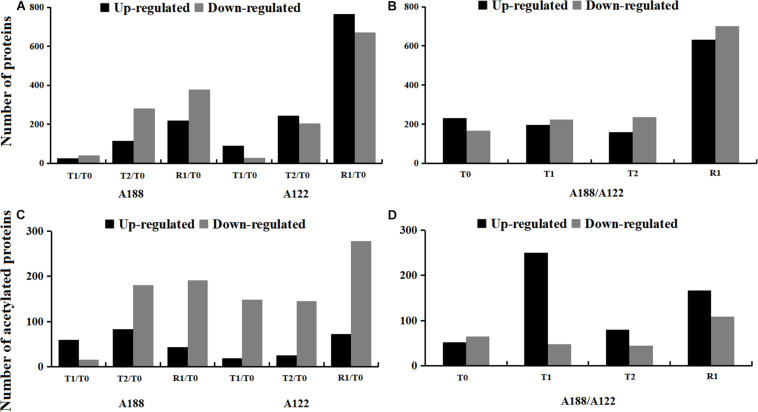
Statistics of differentially expressed proteins/acetylated proteins under cold stress. **(A)** Numbers of differentially expressed proteins in different comparison groups. **(B)** Numbers of differentially expressed proteins between A188 and A122. **(C)** Numbers of differentially expressed acetylated proteins in different comparison groups. **(D)** Numbers of differentially expressed acetylated proteins between A188 and A122. The black and gray columns in the bar chart represent A188 and A122, respectively.

In this study, a total of 3,008 protein species were found to have significantly changed in the two varieties (Supplementary Table 2). Compared to T0 treatment, the number of DEPs increased with the stress and reached their highest number after R1 treatment, but the DEPs of A188 were significantly lower than those of A122 after R1 treatment (Figure 2A). At the same time, the number of downregulated proteins in A188 was always greater than the number of upregulated proteins, whereas proteins in A122 showed the opposite trend. This indicates that there is a significant difference in the protein-expression trend between the cold-resistant variety A188 and the cold-sensitive variety A122 under cold stress. Between these two varieties, the number of DEPs was basically the same after the T0, T1, and T2 treatments, but the number of DEPs reached 1,333 after R1 treatment (Figure 2B). This indicates that the response of these pepper varieties to cold stress is not only reflected in the stress process but also in the impact on the recovery process after the end of cold stress.

### Gene Ontology Term Analysis of DEPs Under Cold Stress

**FIGURE 3:**
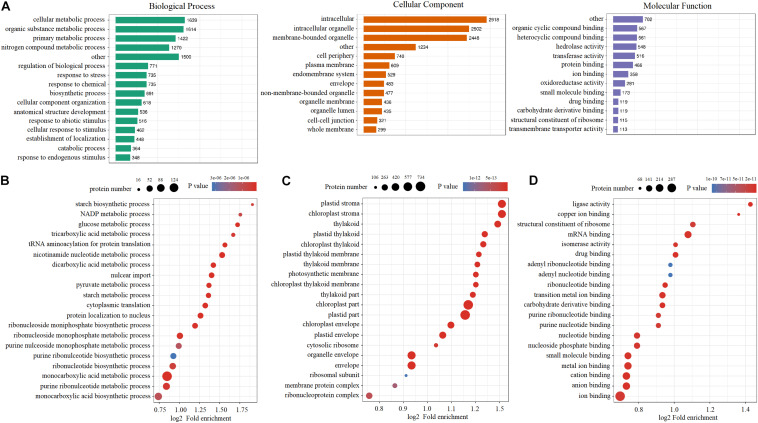
Gene Ontology analysis of quantified and differentially expressed proteins under cold stress. **(A)** GO annotation of quantified and differentially expressed proteins. **(B)** GO enrichment analysis in the biological process. **(C)** GO enrichment analysis in the cellular component. **(D)** GO enrichment analysis in the molecular function.

Gene ontology databases were used to categorize all of the quantified DEPs (Figure 3A). In terms of the number of DEPs, cellular metabolic processes and intracellular and organic cyclic compound binding were predominant in biological processes, cellular components, and molecular function, respectively. To elucidate the functional differences of these proteins, next, the quantified DEPs were analyzed for GO enrichment based on clustering analysis. In the biological process category, many of the DEPs were enriched in the monocarboxylic acid metabolic process and the monocarboxylic acid biosynthetic process (Figure 3B). In the cellular component category, a large number of DEPs were highly enriched in the plastid part, chloroplast part, and organelle envelope (Figure 3C). In terms of molecular function, a large number of DEPs were highly enriched in ion binding, anion binding, and cation binding (Figure 3D).

### Statistics of Acetylation-Related Proteins in Proteome

**FIGURE 4:**
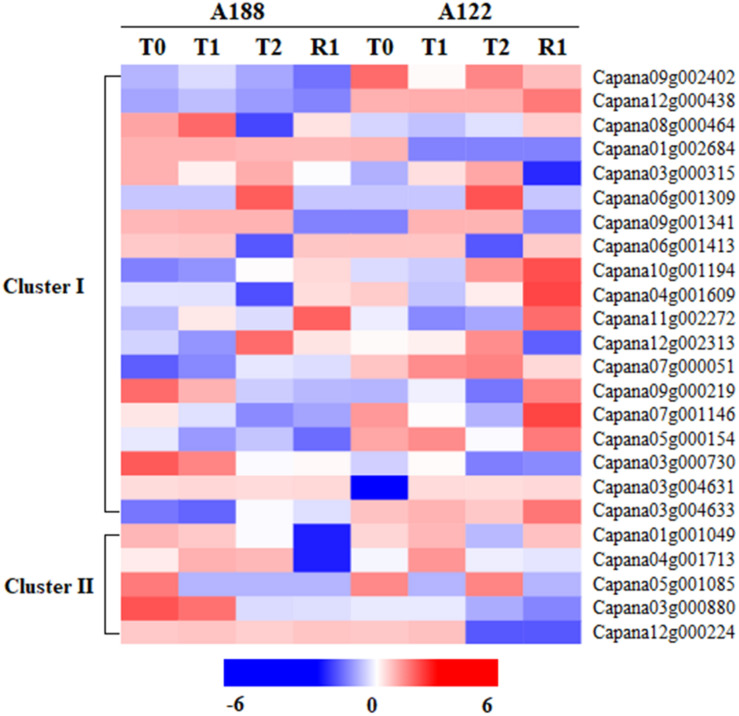
Heat map analysis of acetylation-related proteins in proteomics under cold stress.

In the proteomics data, 99 acylation-related proteins were identified, and 84 of these were quantified (Supplementary Table 3). Among these quantified acylation-related proteins, 24 were closely related to protein acetylation, namely, 19 acetyltransferase proteins (Cluster I) and five deacetylase proteins (Cluster II) (Figure 4). Histone acetyltransferase can participate in transcriptional activation, gene silencing, and other cellular processes, and it plays an important regulatory role (Boycheva et al., 2014). Its protein expression is regulated by abiotic stresses, such as low temperatures (Pavangadkar et al., 2010). Among the 19 acetyltransferase proteins, 10 showed significant changes under cold stress and between varieties differing in sensitivity, but the main change was in the expression of *N*-acetyltransferase proteins.

In the A188 and A122 varieties, the expressions of Capana03g004631 were significantly increased, by 1.512 and 2.079 times in T1, respectively. The expressions of Capana03g000730 significantly decreased by 0.728 and 0.718 times in T2. Additionally, Capana03g004633, Capana09g002402, and Capana12g000438 were found to have no significant changes during the cold stress; their expressions in A188 were always significantly lower than in A122. Ling et al. (2006) and Liu et al. (2007) found that, when treated with H_2_O_2_ or cisplatin, the transcriptional activity and the mRNA expression of *N*-acetyltransferase 10 are significantly increased, indicating that *N*-acetyltransferase expression is affected by related stresses. Enhanced production of melatonin by ectopic overexpression of human serotonin *N*-acetyltransferase has been found to play a role in cold resistance in transgenic rice seedlings (Kang et al., 2010). In this study, the changes in *N*-acetyltransferase in response to cold stress in pepper varieties with different cold-tolerance levels suggest that *N*-acetyltransferase may play a more important regulatory role in response to cold stress than histone acetyltransferase.

In terms of deacetylase, the expression of Capana04g001713 showed no significant changes after the T0, T1, or T2 treatments for either of the varieties, but the expression in A188 was significantly higher after R1 treatment, while the expression in A122 showed no significant changes. The expression of Capana05g001085 and Capana12g000224 was not identified or was low after each treatment of the two varieties, while Capana01g001049 and Capana03g000880 were highly expressed. However, there was no significant difference in these proteins during the whole process or between the two varieties.

Studies have shown that low temperatures can induce a large amount of deacetylase expression in plants (Ma et al., 2013, 2016), and this plays a certain role in improving their cold resistance. To et al. (2011) studied *A. thaliana* and found that HDA6 expression increased significantly after low-temperature treatment, suggesting that HDA6 may play an important role in cold acclimation and cold stress. In this study, the expression of HDA6 (Capana12g000224) was not identified in either of the two varieties after T0 treatment. However, after T1 treatment, the expression of HDA6 was identified in A122 but not A188. This indicates that HDA6 may be involved in the response to cold stress of cold-sensitive pepper varieties.

### Cold Stress Changes the Acetylation Profile in Pepper Seedlings

The acetylation modification of proteins is common in plants, and it is likely to cause extensive modification of various aspects of cell physiology. To further understand the effect of cold stress on the change of acetylation modification in pepper seedlings, the two pepper varieties with different cold sensitivities were analyzed by acetylome. In the acetylome analysis, a total of 9,328 peptides and 4,461 acetylated modified peptides were identified by mass spectrometry. Further analysis found that a total of 2,261 protein groups from 4,574 lysine acetylation sites were identified, of which 3,713 sites in 1,875 protein groups were accurately quantified (Supplementary Table 4). Most of the identified proteins were modified at only one or two sites, and 23.17% of the proteins were modified at more than two sites (Supplementary Figure 5).

### Statistics of Differentially Expressed Acetylated Proteins Under Cold Stress

To screen for differentially expressed acetylated proteins (DEAPs) in this study, proteins containing at least one differentially expressed acetylation site were defined as DEAPs. Under this filtering principle, a total of 768 acetylated protein expressions were found to have significant changes in the two varieties (Supplementary Figure 4B and Supplementary Table 5); among these, 117 were upregulated, and 197 were downregulated (Supplementary Figure 4B). Compared with T0 treatment, only 67 acetylated modified proteins were differentially expressed in A188 after T1 treatment, and most of these proteins showed upregulation. However, the DEAPs after T2 and R1 treatment of A188, and the DEAPs in all treatments of A122 were significantly increased, and these were mainly downregulated (Figure 2C). In the two pepper varieties, the number of DEAPs was the highest after the T1 and R1 treatments, among which the number of DEAPs after T1 treatment reached 285 (Figure 2D). According to the changes in the amount of DEAPs in A188 and A122 after T1 treatment, it can be seen that the sensitivities of the two pepper varieties are notably different, even in the early stages of cold stress. The cold-resistant variety has little stress response to cold, leading to a small number of DEAPs, and these were mainly upregulated; however, the cold-sensitive variety showed a greater stress response to cold; the number of DEAPs changed significantly, and these were mainly downregulated.

### GO Analysis of Co-identified Proteins in Proteome and Acetylome

**FIGURE 5:**
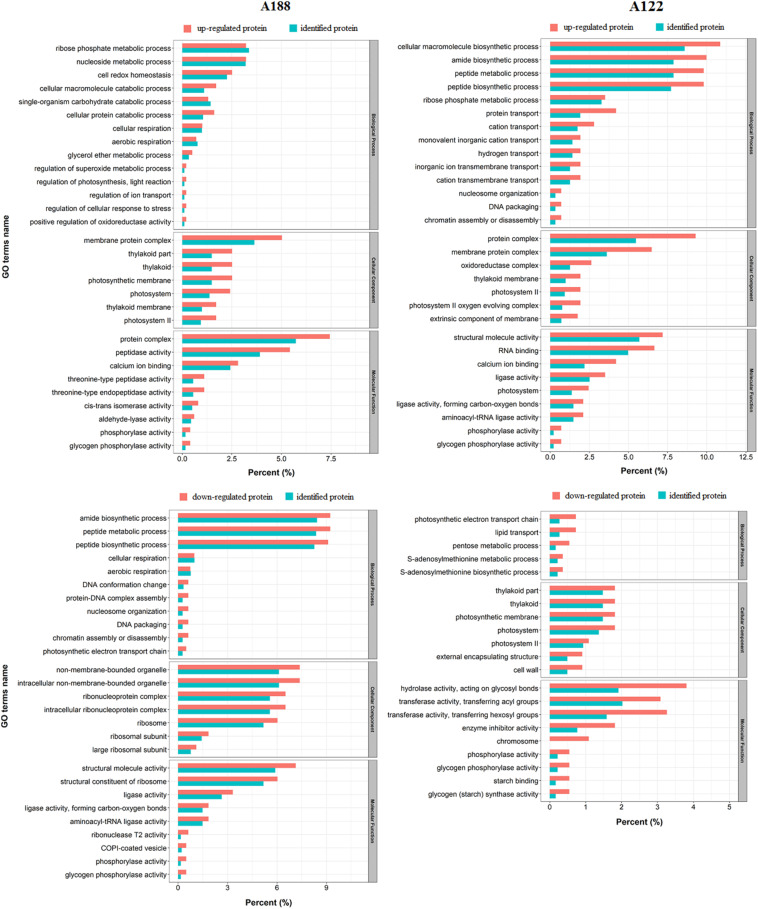
Functional enrichment analysis between the upregulated and downregulated proteins in co-identified proteins. The percentage of differentially expressed proteins indicates the ratio of the mapping proteins to all mapping proteins. The percentage of identified proteins indicates the ratio of the background proteins to all background proteins.

To compare the acetylation with the proteome, all identified acetylated proteins were matched with proteome data. In the results, a total of 1,988 proteins were identified in both the proteome and the acetylome (Supplementary Figure 6 and Supplementary Table 6). To elucidate the functional differences between the upregulated and downregulated proteins in these co-identified proteins, the DEPs were examined for GO enrichment based on clustering analysis (Figure 5). In terms of biological processes, the downregulated proteins in A188 were mainly enriched in the amide biosynthetic process, peptide metabolic process, and peptide biosynthetic process categories, while many of the upregulated proteins in A122 were enriched in these categories. In the cellular component category, many of the upregulated proteins in both A188 and A122 were enriched in the membrane protein complex. In the molecular function category, in A188, many of the upregulated proteins were enriched in protein complex and peptidase activity; downregulated proteins were mainly enriched in structural molecule activity and structural constituents of ribosomes. In A122, many of the upregulated proteins were enriched in structural molecule activity and RNA binding, while many of the downregulated proteins were enriched in hydrolase activity, acting on glycosyl bonds and transferase activity, transferring hexosyl groups. Clearly, the amide biosynthetic process, peptide metabolic process, peptide biosynthetic process, and structural molecule activity categories in the GO enrichment analysis showed large differences between A188 and A122. It could be suggested that the proteins and acetylated proteins in these categories may play important roles in the cold resistance of pepper plants.

### Motif Analysis of Lysine-Acetylated Sites

**FIGURE 6:**
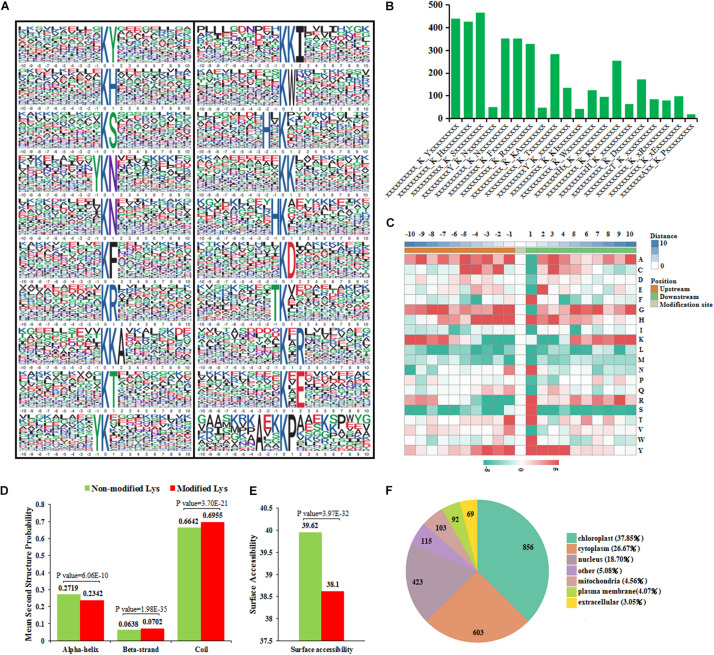
Functional bioinformational analysis of acetylated proteins. **(A)** Sequence motif analysis of acetylated sites. **(B)** The number of identified peptides containing acetylated sites in each motif. **(C)** The relative abundance of amino acid residues flanking the acetylated sites represented by an intensity map. **(D)** Comparison of non-modified and modified-acetylated proteins amino acids in protein secondary structures. **(E)** Comparison of non-modified and modified-acetylated proteins amino acids in protein surface accessibility. **(F)** Distribution of the acetylated proteins in subcellular compartments.

Twenty acetylation motifs were defined on 3,934 unique sites, accounting for 86.01% of the total lysine-acetylated sites, using the MoMo software package (Figure 6A and Supplementary Table 7). These motifs exhibited different abundances, and motifs ^∗^KS^∗^, ^∗^KY^∗^, and ^∗^KH^∗^ occupied the highest proportions of all the identified peptides; ^∗^AxxKP^∗^ occupied the lowest proportion in these motifs (Figure 6B). This indicates that the lysine residues around some alkaline residues (S, Y, or H) may be more easily acetylated under cold stress.

There is increasing evidence that suggests that different species show distinct preferences for amino acid residues at specific positions surrounding acetylated lysines (Pan et al., 2014). According to the heat map of the amino acid compositions surrounding the acetylation sites, histidine (H) and tyrosine (Y) were significantly overrepresented in positions −3 to +3; other residues, such as phenylalanine (F) and serine (S), were highly presented in position +1 (Figure 6C). Previous studies have found that three motifs common in pepper (^∗^KY^∗^, ^∗^KH^∗^, and ^∗^KF^∗^) also exist in rice (Hartl et al., 2017) and strawberry (Smith-Hammond et al., 2014). This indicates that the preferences for amino acid distribution present similarities among different species, although some other distinct amino acid residues were also present in these species.

### Secondary Structure Analysis of Acetylated Proteins

To determine whether acetylated lysine occurs frequently within particular structures in proteins, the probabilities of different secondary structures near acetylated lysine sites were compared with the secondary structure probabilities of non-modified lysine sites on the proteins identified in this study. The results show that the possibility of α-helix occurring in non-modified lysine are higher than in modified lysine, while the possibility of coiling occurring in modified lysine was higher than in non-modified lysine (Figure 6D). A total of 69.55% of modified lysine was distributed in regions predicted to be disordered (coiled) proteins. In addition, 30.48% of modified lysine was distributed in ordered regions. This result was highly similar to the distribution in a silkworm (Nie et al., 2015) and rice (Nallamilli et al., 2014). This indicates that the acetylated modifications also tend to occur in the unstructured regions (coils) in pepper proteins. The surface accessibility of the acetylated lysine was also analyzed. The results show that 39.62% of non-modified lysine and 38.10% of the modified lysine, respectively, were located on the protein surface (Figure 6E). Therefore, lysine acetylation is likely to slightly affect the surface properties of modified proteins in pepper.

### Subcellular Localization Analysis of Acetylated Proteins

During the whole process of cold-stress treatment, subcellular distribution predictions showed that acetylated proteins in A188 and A122 pepper leaves were distributed predominantly in the chloroplast, cytoplasm, and nucleus. In contrast, there were relatively few acetylated proteins located in the mitochondria, plasma membrane, or extracellular space (Figure 6F). Compared with the T0 treatment, after the T1 and R1 treatments, A122 showed more DEAPs in the chloroplasts and cytoplasm than A188. In addition, between the two varieties, the number of DEAPs in the chloroplasts and cytoplasm after the T1 and R1 treatments was also the highest (Supplementary Figure 7). This suggests that cold stress mainly affects the abundance of acetylated proteins located in the chloroplasts and cytoplasm of pepper leaves.

### Clustering Analysis of Acetylated Proteins

**FIGURE 7:**
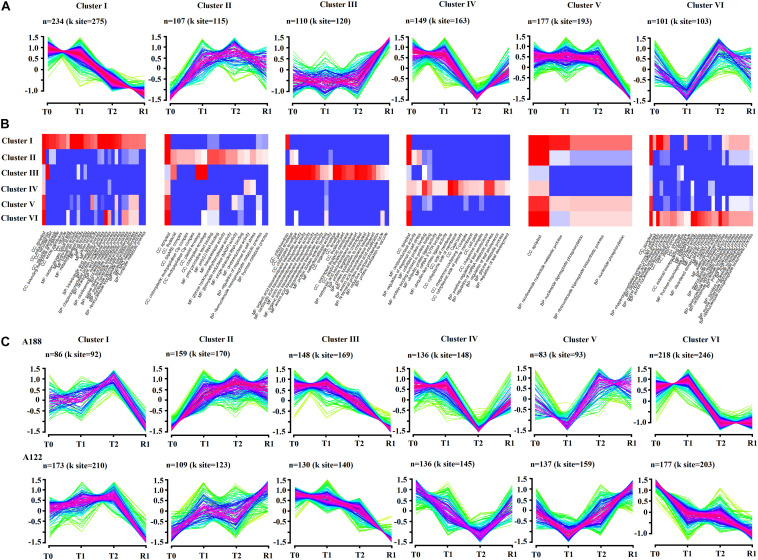
Dynamic acetylome remodeling of peppers during cold stress and recovery stages. **(A)** Unsupervised clustering of acetylome dynamics revealed top six clusters with distinct protein expression profiles. n represents the number of acetylated proteins per cluster. **(B)** Gene ontology enrichment analysis of each cluster. Representative biological process (BP), cellular components (CC), and molecular function (MF) that are overrepresented in one of the clusters are visualized. **(C)** Unsupervised clustering of acetylome dynamics revealed top six clusters with distinct protein expression profiles in A188 and A122, respectively. n represents the number of acetylated proteins per cluster.

To obtain an unbiased view of the acetylated proteome expression dynamics under cold stress, we performed unsupervised fuzzy clustering of all DEAPs. Through this analysis, six trend clusters with the largest number of acetylated proteins were obtained (Figure 7A). Furthermore, GO functional annotation analysis was also carried out for the DEAPs in these clusters (Figure 7B). The results showed that all of the acetylated proteins in Clusters II and III were upregulated. The proteins in Cluster II were mainly associated with apoplasts, poly-pyrimidine tract binding, and poly (U) RNA binding, while the proteins in Cluster III were mainly involved in the terms associated with the transmembrane transporter activity and envelope. Furthermore, some acetylated proteins in Clusters II and III were found to be enriched in thylakoids, plastid envelope, and chloroplast envelope. The proteins in Cluster IV were mainly involved in unfolded protein binding, positive regulation of leaf senescence, and regulation of miRNA metabolic process. The proteins in Cluster VI were associated with apoplast, nicotinamide adenine dinucleotide phosphate (NADP) binding, and fructose-bisphosphate aldolase activity. Acetylated proteins in Clusters I and V were both downregulated under cold stress. The proteins in Cluster I were mainly involved in apoplasts, mitochondria, and some terms associated with metabolic processes. However, proteins in Cluster V were mainly associated with apoplasts. It can be seen from this clustering trend that acetylated proteins in Clusters I, II, V, and VI were clustered into apoplasts in large quantities, indicating that significant changes in apoplasts in pepper leaves were expressed more under cold stress.

To explore the reasons for the difference in cold resistance of the two pepper varieties, trend clustering analyses of A188 and A122 were conducted, and it was found that the top-six changes (those with the largest numbers) in the clusters for the two pepper varieties had the same overall trend, but there were still significant differences between the two varieties (Figure 7C). The trends in the change of acetylated proteins in Clusters II and V were opposite in treatments T2 and R1. The variation trend in Clusters IV and VI showed significant differences between treatments T0 and T1; the acetylated protein expression abundance of A188 was basically unchanged, while that of A122 was significantly downregulated. The difference in Clusters II, IV, V, and VI indicated that the difference in the expression trend of acetylated proteins in treatments T1 and R1 was an important reason for the significant difference in the resistance to cold stress between the two peppers. In the early stages of cold stress (T1 treatment), the expression of some important proteins in A122, which has weak resistance to low-temperature stress, was significantly downregulated; this may weaken its resistance to cold stress and deepen the damage caused by it. However, after a long period of cold stress (R1 treatment), the cold-resistant pepper A188 suffered less damage, and a large number of proteins showed little change, while proteins in the cold-sensitive pepper A122 were significantly upregulated to repair stress damage.

### KEGG Analysis of Acetylated Proteins

Lysine acetylation has been shown to play a major role in metabolism regulation in eukaryotes (Walley et al., 2018). In this study, 397 identified acetylated proteins were involved in 93 different metabolic pathways (Supplementary Table 8). Of these metabolic pathways, 21 identified more than 10 acetylated proteins. Among these, 49 acetylated proteins were involved in ribosomes, 35 were involved in glycolysis/gluconeogenesis, 32 were involved in glyoxylate and dicarboxylate metabolism, 31 were involved in carbon fixation in photosynthetic organisms, 23 were involved in protein processing in endoplasmic reticulum, 22 were involved in the pentose phosphate pathway and glutathione metabolism, and 19 were involved in oxidative phosphorylation and photosynthesis. Almost all the proteins involved in the basic metabolic pathway were identified to be acetylated, indicating the role of reversible lysine acetylation in regulating and controlling cellular protein activity at the PTM level during cold stress.

### Photosynthesis

**FIGURE 8:**
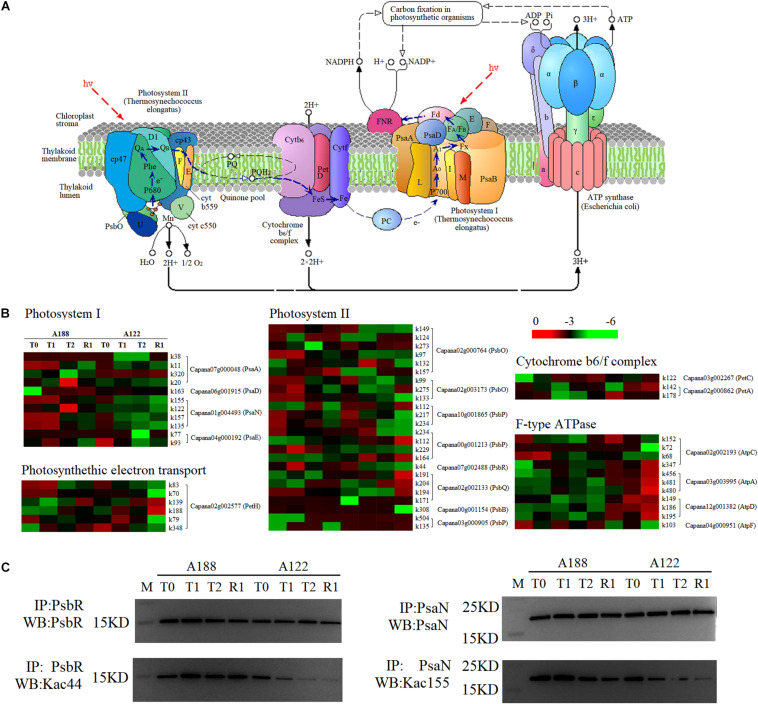
Comparative analysis of acetylated proteins involved in the photosynthesis pathway. **(A)** Schematic illustration of the photosynthesis pathway. **(B)** Heat map analysis of acetylated proteins involved in the photosynthesis pathway. **(C)** Western blotting analysis of photosystem II 10 kDa polypeptide (PsbR) acetylation and photosystem I reaction center subunit N (PsaN). PsaA, photosystem I P700 chlorophyll a apoprotein A1; PsaD, photosystem I reaction center subunit II; PsaN, photosystem I reaction center subunit N; PsaE, Photosystem I reaction center subunit IV A; PsbO, oxygen-evolving enhancer protein 1; PsbP, oxygen-evolving enhancer protein 2; PsbR, photosystem II 10 kDa polypeptide; PsbQ, oxygen-evolving enhancer protein 3-2; PsbB, photosystem II CP47 chlorophyll apoprotein; PetC, cytochrome b6-f complex iron-sulfur subunit; PetA, apocytochrome f; PetH, Ferredoxin–NADP reductase; AtpA, ATP synthase subunit alpha; AtpC, ATP synthase gamma chain; AtpD, ATP synthase delta chain; AtpF, ATP synthase subunit b.

Light functions in photosynthesis to produce “assimilatory power” (ATP and NADPH) to push the dark reaction, and the process is carried out by photosystem I (PS I), photosystem II (PS II), and light-harvesting pigments in the thylakoid membrane. Under cold stress, other substances, such as peroxiredoxins, H_2_O_2_, and metabolic intermediates, will be produced due to the imbalance caused by excessive energy being captured by the chlorophyll antenna complex (Huner et al., 2012). When suffering cold stress, proteins in the photosynthesis pathway are modified by different degrees of acetylation. A total of 111 acetylation modification sites were identified from 19 acetylated proteins in the photosynthetic pathway. Among these, 55 sites showed significant differences in the level of acetylation modification (Figure 8 and Supplementary Table 9).

Photosystem II is responsible for absorbing light energy and using it to split H_2_O, producing protons and releasing O_2_. Cold stress and other environmental factors can easily lead to reduced PS II transport capacity by changing the protein expression pattern (Huner et al., 2012; Lamers et al., 2020). Studies have shown that the oxygen-evolving enhancer protein 1 (PsbO) complex is the most closely related peripheral protein, and this plays the role of stabilizing manganese clusters while playing an auxiliary role in maintaining the oxygen release reaction on the chloroplast thylakoid membrane (Hasni et al., 2015). Removal of this protein results in both the loss of oxygen-evolving activity and a reduction in the number of manganese ions bound to the thylakoid membrane (Mayfield et al., 1987). A deficiency in the PS II 10-kDa polypeptide (PsbR) protein reduces the expression levels of oxygen-evolving enhancer protein 2 (PsbP), oxygen-evolving enhancer protein 3-2 (PsbQ), and D2 proteins in PS II, resulting in the decrease of the oxygen precipitation rate and the reoxidation of quinone (Allahverdiyeva et al., 2013).

Acetylation can have a range of biochemical and biological effects; acetylation of LHCB1 and LHCB2 appears to influence LHC attachment to PS II complexes (Wu et al., 2011). Liu et al. (2019) found that the acetylation levels of PsaH2, LHCB6, and LHCA1 at high temperatures were downregulated in grape leaves. In this study, the acetylation levels of PsbO (K99 and K133 of Capana02g003173, K97, and K273 of Capana02g000764) and PsbR (K44 of Capana07g002488) in the PS II of A188 were significantly higher than those of A122 at the initial stage of cold stress (2 h); the acetylation levels of PsbO (K149 of Capana02g000764, K133 of Capana02g003173) and PsbR (K44 of Capana07g002488) were still significantly higher than those of A122, following 1 h of recovery after 72 h of cold stress. This indicates that acetylation of the PsbO and PsbR proteins could regulate the response of leaves to cold stress and promote the activity of these proteins in PS II with the increase of their modification level. This will accelerate water cleavage, release oxygen, and alleviate the photoinhibition effect of cold stress on PS II. To validate the acetylation events in PsbR (K44 of Capana07g002488), we carried out western blotting analysis of the whole cell lysate under different cold conditions, using a pan anti-acetylation antibody and an anti-PsbR-K44 acetyl antibody (Figure 8C). Our findings confirmed PsbR (K44 of Capana07g002488) acetylation through immunoprecipitation, and the acetylation levels remained consistent with the lysine acetylome results.

Although PS II is considered to be the primary site of photoinhibition, it is the component most sensitive to low temperatures in photosynthetic apparatus (Gerganova et al., 2016). However, studies have found that under low-temperature photoinhibition, PS I rather than PS II is the primary action site of photoinhibition in higher plants, and the photoinhibition of PS I is the main reason for photosynthetic decline of cold-sensitive plants under cold stress (Yang et al., 2017). Cold stress can inhibit the activity of plant carbon dioxide fixation-related enzymes, thus inhibiting the rate of carbon assimilation, leading to the excessive accumulation of reducing force NADPH on the PS I receptor side, causing excessive reduction (Golbeck, 1987), promoting the generation of hydroxyl radicals, and ultimately accelerating the photoinhibition of PS I (Huang et al., 2018). In this study, the acetylation level of PS I reaction center subunit N (PsaN, k155 of Capana01g004493) protein of cold-resistant material A188 was always significantly higher than that of A122 under cold stress and recovery after cold stress, while other subunit proteins in PS I showed no significant differences. PsaN, a subunit that mediates LHCII energy delivery to the PS I core in plants and green algae (Vanselow et al., 2009), plays an important role in drawing from plastocyanin to P700 efficient electron transport. In the absence of PsaN, the second-order rate constant for plastocyanin oxidation decreases to about 60% (Haldrup et al., 1999). This indicates that the increase of the PsaN (k155 of Capana01g004493) acetylation modification level in the light system PS I may promote the energy transfer in PS I, which plays an important role in alleviating the photoinhibition of PS I under cold stress. To validate the acetylation events in PsaN (k155 of Capana01g004493), we also carried out western blotting analysis of the whole cell lysate under different cold conditions, using a pan anti-acetylation antibody and an anti-PsaN-K155 acetyl antibody (Figure 8C). Our findings confirmed PsaN (k155 of Capana01g004493) acetylation through immunoprecipitation, and the acetylation levels remained consistent with the lysine acetylome results.

### Carbon Fixation in Photosynthetic Organisms

**FIGURE 9:**
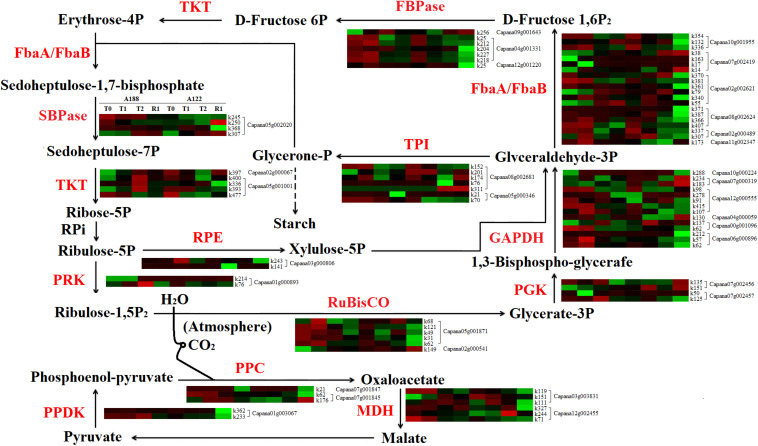
Comparative analysis of acetylated proteins involved in carbon fixation in photosynthetic organisms. FBPase, fructose-1,6-bisphosphatase; TKT, Transketolase; SBPase, sedoheptulose-1,7-bisphosphatase; PRK, phosphoribulokinase; RPE, ribulose-phosphate 3-epimerase; PPDK, pyruvate, phosphate dikinase; TPI, triosephosphate isomerase; GAPDH, glyceraldehyde-3-phosphate dehydrogenase; RuBisco, ribulose bisphosphate carboxylase; PPC, phosphoenolpyruvate carboxylase; MDH, malate dehydrogenase; FbaA/FbaB, fructose-bisphosphate aldolase. PGK, phosphoglycerate kinase.

When suffering cold stress, most enzyme proteins in the “carbon fixation in photosynthetic organisms” pathway were also modified by different degrees of acetylation. A total of 163 acetylation modification sites were identified from 31 acetylated proteins in the carbon fixation in photosynthetic organism pathway, of which 82 sites showed significant differences in the level of acetylation modification (Supplementary Table 10 and Figure 9). It was found that the acetylation level of proteins in the carbon fixation in photosynthetic organism pathway of the two pepper varieties mainly decreased across the whole period of cold stress and recovery.

Low temperatures inhibited sucrose synthesis in the cytoplasm and reduced the circulation between the cytoplasm and chloroplast of inorganic phosphate released by sucrose synthesis (Hurry et al., 2000). This, in turn, hindered the synthesis of the ATP required for ribulose-1,5-phosphate reconstruction, resulting in the failure of normal fixation of CO_2_. The activity of fructose-1,6-bisphosphatase (FBPase) in metabolic pathways also affects starch synthesis and carbon metabolism balance (Rojas-González et al., 2015; Gütle et al., 2016). Studies have found that low-temperature stress can reduce the activity of FBPase in tomatoes (Hutchison et al., 2000) and woody plants (Allen and Ort, 2001). In this study, there were no significant changes in the protein abundance of ribulose bisphosphate carboxylase (Rubisco) and FBPase under cold stress. However, in the initial stages of cold stress (T1 treatment), the acetylation levels of FBPase (k212, k218, and k227 of Capana04g001331) in A188 were significantly higher than those in A122 (Figure 9). This indicates that maintaining high levels of acetylation of FBPase in the early stages of cold stress may be an important molecular regulatory mechanism for enhancing the cold resistance of pepper plants.

There are also sedoheptulose-1,7-bisphosphatases (SBPases) similar to FBPases in plants, which have the same origin but different functions in the process of evolution. SBPase is a unique and key enzyme, and its activity is highly regulated; it controls the carbon circulation in the Calvin cycle process (Köhler et al., 2017). Studies have shown that SBPase activity is affected by the reduced reduction capacity of chloroplast matrix at low temperatures (Wise, 1995), and a high level of SBPase activity under low-temperature stress is beneficial to the photosynthesis and carbon fixation of tomato plants and resistance to low-temperature-induced oxidative stress—thus, enhancing the resistance of tomatoes to low-temperature stress (Ding et al., 2017). Glyceraldehyde-3-phosphate dehydrogenase (GAPDH) in chloroplasts plays a central role in the Calvin cycle of CO_2_ assimilation through specific binding of NADPH (McFarlane et al., 2019). The promoter of DbGAPDH in Chlorophyta (*Dunaliella bardawil*) contained six obvious regulatory elements, including an aerobic response element, a light regulatory element, and a cold-stress regulatory element, indicating that DbGAPDH has a certain regulatory ability for cold stress (Lao et al., 2012); GAPDH and phosphoribulokinase (PRK) synergistically regulate the Calvin cycle by forming a reversible PRK/GAPDH/CP12 multi-enzyme complex with the chloroplast intrinsic protein CP12 (Howard et al., 2011).

In this study, the acetylation levels of SBPase, PRK, and GAPDH were significantly higher in A188 than A122 after treatments T1 and T2. Among these, the acetylation levels of SBPase (K245 of Capana05g002020), PRK (K76 of Capana01g000893), and triosephosphate isomerase (TPI, K70 of Capana05g000346) were always significantly higher in A188 than in A122, while the acetylation levels of GAPDH (k57 and k62 of Capana06g000896, k130 of Capana04g0059, k98 of Capana12g000555) and GAPDH (k212 of Capana06g000896, k107 and k178 of Capana12g000555) were increased after treatments T1 and T2, respectively. However, the abundance of these proteins did not change significantly overall under cold stress. Liu et al. (2019) also established that acetylation had a greater effect on the Calvin cycle than other modifications. This indicates that the acetylation levels of SBPase, PRK, and GAPDH are positively correlated with their enzyme activity; the higher the level of their acetylation modification, the stronger the enzyme activity and the higher the carbon assimilation capacity, and this, therefore, reduces photoinhibition damage. Moreover, the increase of the TPI (k70 of Capana05g000346) acetylation level in A188 is helpful to catalyze the formation of glyceraldehyde 3-phosphate into glycerol phosphate and accelerate the cycle speed of the carbon assimilation and the regeneration stage. Therefore, we consider that the decrease of the acetylation levels of key carbon assimilation enzymes, such as Rubisco, FBPase, SBPase, GAPDH, PRK, and TPI under cold stress, is an important reason for the decreases in carbon assimilation capacity and photosynthetic efficiency. Enhancing the acetylation levels of key carbon assimilation enzymes can thus promote the carbon assimilation pathway to fix CO_2_ and produce more sucrose, which then forms fructan and other substances to accumulate in mesophyll cells (Savitch et al., 2002), thus effectively enhancing the ability of pepper plants to resist cold stress.

## Conclusion

In summary, a total of 6,213 proteins and 4,574 acetylated protein sites were identified by proteome and lysine acetylome analysis in this study. Further analysis found that a total of 1,988 proteins were identified in both the proteome and acetylome, and the functional differences in these co-identified proteins were elucidated through GO enrichment. The dynamic changes in the acetylated proteins in photosynthesis and the carbon fixation in the photosynthetic organism pathway in pepper under low-temperature stress were further analyzed. It was found that acetylation of the PsbO and PsbR proteins in the PS II light system and the PsaN protein in the PS I light system could regulate the response of pepper leaves to cold stress, and the acetylation levels of Rubisco, FBPase, SBPase, GAPDH, PRK, and TPI of key carbon assimilation enzymes decreased, leading to decreases in carbon assimilation capacity and photosynthetic efficiency, reducing the cold tolerance of pepper leaves. This study greatly expands the catalog of lysine acetylation substrates and sites in Solanaceae crops and provides new insights for posttranslational modification studies.

## Data Availability Statement

The original contributions presented in the study are publicly available. This data can be found here: the mass spectrometry Proteome and Acetylome data both have been deposited to the ProteomeXchange Consortium (https://www.ebi.ac.uk/pride/archive/) via the PRIDE partner repository with the dataset identifier PXD021024. Data generated during analysis are included in the manuscript as Supplementary Material.

## Author Contributions

ZL: formal analysis and writing-original draft. XZ and LO: funding acquisition. ZL, JS, and WM: investigation. ZL, BY, FT, and HS: methodology. XD and XZ: project administration. ZZ and WC: resource. XZ and LO: supervision. LO: writing-review and editing. All authors read and approved the final manuscript.

## Conflict of Interest

WM was employed by Hunan Xiangyan Seed Industry Co., Ltd. The remaining authors declare that the research was conducted in the absence of any commercial or financial relationships that could be construed as a potential conflict of interest.

## Publisher’s Note

All claims expressed in this article are solely those of the authors and do not necessarily represent those of their affiliated organizations, or those of the publisher, the editors and the reviewers. Any product that may be evaluated in this article, or claim that may be made by its manufacturer, is not guaranteed or endorsed by the publisher.

## Footnotes

1
http://www.ebi.ac.uk/GOA/

2
http://www.ebi.ac.uk/interpro/

3
http://geneontology.org/

4
http://meme-suite.org/tools/momo

5
http://www.genscript.com/psort/wolf_psort.html

## Supplementary Material

Supplementary Figure 1 Phenotypic changes in the two pepper varieties during cold stress and recovery.

Supplementary Figure 2 Western blotting with antibodies. **(A)** Anti-crotonyllysine. **(B)** Anti-2-hydroxyisobutyryllysine. **(C)** Anti-succinyllysine. **(D)** Anti-acetyllysine.

Supplementary Figure 3 Transmission electron microscope analysis of A188 (cold-resistant variety) and A122 (cold-sensitive variety) under cold stress and recovery stages.

Supplementary Figure 4 Venn diagrams of samples. **(A)** Numbers of proteins identified in each treatment. **(B)** Numbers of differentially expressed acetylation proteins under cold stress.

Supplementary Figure 5 Statistics on the number of acetylation sites in proteins.

Supplementary Figure 6 Statistics of co-identified proteins in proteome and acetylome.

Supplementary Figure 7 Subcellular localization analysis of acetylated proteins.

Supplementary Table 1 Pepper protein intensity data under cold stress.

Supplementary Table 2 Statistics of differentially expressed proteins under cold stress.

Supplementary Table 3 Statistics of acetylation-related proteins in proteome data.

Supplementary Table 4 Pepper-acetylated protein intensity data under cold stress.

Supplementary Table 5 Statistics of differentially expressed acetylated proteins under cold stress.

Supplementary Table 6 A list of co-identified proteins in proteome and acetylome.

Supplementary Table 7 Motif annotation of acetylated proteins under cold stress.

Supplementary Table 8 A list of the identified acetylated proteins in KEGG pathways.

Supplementary Table 9 A list of acetylation modification sites identified in the photosynthetic pathway.

Supplementary Table 10 A list of acetylation modification sites identified in the carbon fixation in the photosynthetic organism pathway.

## References

[B1] Airaki M. Leterrier M. Mateos R. M. Valderrama R. Chaki M. Barroso J. B. (2012). Metabolism of reactive oxygen species and reactive nitrogen species in pepper (*Capsicum annuum* L.) plants under low temperature stress. *Plant Cell Environ.* 35 281–295. 10.1111/j.1365-3040.2011.02310.x 21414013

[B2] Allahverdiyeva Y. Suorsa M. Rossi F. Pavesi A. Kater M. M. Antonacci A. (2013). *Arabidopsis* plants lacking PsbQ and PsbR subunits of the oxygen-evolving complex show altered PSII super-complex organization and short-term adaptive mechanisms. *Plant J.* 75 671–684. 10.1111/tpj.12230 23647309

[B3] Allen D. J. Ort D. R. (2001). Impacts of chilling temperatures on photosynthesis in warm-climate plants. *Trends Plant Sci.* 6 36–42. 10.1016/s1360-1385(00)01808-2 11164376

[B4] Aroca R. Vernieri P. Irigoyen J. J. Sánchez-Dıaz M. Tognoni F. Pardossi A. (2003). Involvement of abscisic acid in leaf and root of maize (*Zea mays* L.) in avoiding chilling-induced water stress. *Plant Sci.* 165 671–679. 10.1016/S0168-9452(03)00257-7

[B5] Aza-González C. Núñez-Palenius H. G. Ochoa-Alejo N. (2011). Molecular biology of capsaicinoid biosynthesis in chili pepper (Capsicum spp.). *Plant Cell Rep.* 30 695–706. 10.1007/s00299-010-0968-8 21161234

[B6] Boycheva I. Vassileva V. Iantcheva A. (2014). Histone acetyltransferases in plant development and plasticity. *Curr. Genomics* 15:28. 10.2174/138920291501140306112742 24653661 PMC3958957

[B7] Ciacka K. Krasuska U. Otulak K. K. Gniazdowska A. (2019). Dormancy removal by cold stratification increases glutathione and S-nitrosoglutathione content in apple seeds. *Plant Physiol. Biochem.* 138 112–120. 10.1016/j.plaphy.2019.02.026 30861401

[B8] Ding F. Wang M. Zhang S. (2017). Overexpression of a Calvin cycle enzyme SBPase improves tolerance to chilling-induced oxidative stress in tomato plants. *Sci. Hortic.* 214 27–33. 10.3389/fpls.2020.565701 33414794 PMC7783158

[B9] Fang X. Chen W. Zhao Y. Ruan S. Zhang H. Yan C. (2015). Global analysis of lysine acetylation in strawberry leaves. *Front. Plant Sci.* 6:739. 10.3389/fpls.2015.00739 26442052 PMC4569977

[B10] Finkemeier I. Laxa M. Miguet L. Howden A. J. Sweetlove L. J. (2011). Proteins of diverse function and subcellular location are lysine acetylated in *Arabidopsis*. *Plant Physiol.* 155 1779–1790. 10.1104/pp.110.171595 21311031 PMC3091095

[B11] Gerganova M. Popova A. V. Stanoeva D. Velitchkova M. (2016). Tomato plants acclimate better to elevated temperature and high light than to treatment with each factor separately. *Plant Physiol. Biochem.* 104 234–241. 10.1016/j.plaphy.2016.03.030 27038602

[B12] Gharechahi J. Alizadeh H. Naghavi M. R. Sharifi G. (2014). A proteomic analysis to identify cold acclimation associated proteins in wild wheat (*Triticum urartu L*.). *Mol. Biol. Rep.* 41 3897–3905. 10.1007/s11033-014-3257-8 24535272

[B13] Golbeck J. H. (1987). Structure, function and organization of the Photosystem I reaction center complex. *Biochim. Biophys. Acta Rev. Bioenerg.* 895 167–204. 10.1016/s0304-4173(87)80002-2 3333014

[B14] Gütle D. D. Roret T. Müller S. J. Couturier J. Lemaire S. D. Hecker A. (2016). Chloroplast FBPase and SBPase are thioredoxin-linked enzymes with similar architecture but different evolutionary histories. *Proc. Natl. Acad. Sci. U. S. A.* 113 6779–6784. 10.1073/pnas.1606241113 27226308 PMC4914176

[B15] Haldrup A. Naver H. Scheller H. V. (1999). The interaction between plastocyanin and photosystem I is inefficient in transgenic *Arabidopsis* plants lacking the PSI-N subunit of photosystem. *Plant J.* 17 689–698. 10.1046/j.1365-313x.1999.00419.x 10230065

[B16] Hart G. W. Ball L. E. (2013). Post-translational modifications: a major focus for the future of proteomics. *Mol. Cell. Proteomics* 12 3443–3443. 10.1074/mcp.E113.036491 24277940 PMC3861697

[B17] Hartl M. Füßl M. Boersema P. J. Jost J. O. Kramer K. Bakirbas A. (2017). Lysine acetylome profiling uncovers novel histone deacetylase substrate proteins in *Arabidopsis*. *Mol. Syst. Biol.* 13:949. 10.15252/msb.20177819 29061669 PMC5658702

[B18] Hasni I. Yaakoubi H. Hamdani S. Tajmir-Riahi H. A. Carpentier R. (2015). Mechanism of interaction of Al3+ with the proteins composition of photosystem II. *PLoS One* 10:e0120876. 10.1371/journal.pone.0120876 25806795 PMC4373732

[B19] Howard T. P. Lloyd J. C. Raines C. A. (2011). Inter-species variation in the oligomeric states of the higher plant Calvin cycle enzymes glyceraldehyde-3-phosphate dehydrogenase and phosphoribulokinase. *J. Exp. Bot.* 62 3799–3805. 10.1093/jxb/err057 21498632 PMC3134340

[B20] Hu Y. Zhang L. Zhao L. Li J. He S. Zhou K. (2011). Trichostatin A selectively suppresses the cold-induced transcription of the ZmDREB1 gene in maize. *PLoS One* 6:e22132. 10.1371/journal.pone.0022132 21811564 PMC3141014

[B21] Huang W. Tikkanen M. Zhang S. B. (2018). Photoinhibition of photosystem I in Nephrolepis falciformis depends on reactive oxygen species generated in the chloroplast stroma. *Photosynth. Res.* 137 129–140. 10.1007/s11120-018-0484-1 29357086

[B22] Huner N. Dahal K. Hollis L. Bode R. Rosso D. Krol M. (2012). Chloroplast redox imbalance governs phenotypic plasticity: the “grand design of photosynthesis” revisited. *Front. Plant Sci.* 3:255. 10.3389/fpls.2012.00255 23230444 PMC3515967

[B23] Hurry V. Strand A. Furbank R. Stitt M. (2000). The role of inorganic phosphate in the development of freezing tolerance and the acclimatization of photosynthesis to low temperature is revealed by the pho mutants of *Arabidopsis thaliana*. *Plant J.* 24 383–396. 10.1046/j.1365-313x.2000.00888.x 11069711

[B24] Hutchison R. S. Groom Q. Ort D. R. (2000). Differential effects of chilling-induced photooxidation on the redox regulation of photosynthetic enzymes. *Biochemistry* 39 6679–6688. 10.1021/bi0001978 10828986

[B25] Kang K. Lee K. Park S. Kim Y. S. Back K. (2010). Enhanced production of melatoninbyectopic overexpression of human serotonin N-acetyltransferase plays a role in cold resistance in transgenic rice seedlings. *J. Pineal Res.* 49 176–182. 10.1111/j.1600-079X.2010.00783.x 20586889

[B26] Köhler I. H. Ruiz-Vera U. M. VanLoocke A. (2017). Expression of cyanobacterial FBP/SBPase in soybean prevents yield depression under future climate conditions. *J. Exp. Bot.* 68 715–726. 10.1093/jxb/erw435 28204603 PMC5441901

[B27] Kosová K. Vítámvás P. Planchon S. Renaut J. Vanková R. Prášil I. T. (2013). Proteome analysis of cold response in spring and winter wheat (*Triticum aestivum*) crowns reveals similarities in stress adaptation and differences in regulatory processes between the growth habits. *J. Proteome Res.* 12 4830–4845. 10.1021/pr400600g 24047233

[B28] Lamers J. Meer T. Testerink C. (2020). How plants sense and respond to stressful environments. *Plant Physiol.* 182 1624–1635. 10.1104/pp.19.01464 32132112 PMC7140927

[B29] Lao Y. M. Lu Y. Jiang J. G. Luo L. X. (2012). Six regulatory elements lying in the promoter region imply the functional diversity of chloroplast GAPDH in Duanliella bardawil. *J. Agric. Food Chem.* 60 9211–9220. 10.1021/jf302659z 22906227

[B30] Li B. Carey M. Workman J. L. (2007). The role of chromatin during transcription. *Cell* 128 707–719. 10.1016/j.cell.2007.01.015 17320508

[B31] Ling Y. Liu H. J. Hou L. Zhang B. (2006). Enhanced expression of halp gene confers cellular resistance to H2O2 induced senescence. *Chin. Med. Sci. J.* 21 1–5. 16615275

[B32] Liu G. T. Jiang J. F. Liu X. N. Jiang J. Z. Sun L. Duan W. (2019). New insights into the heat responses of grape leaves via combined phosphoproteomic and acetylproteomic analyses. *Hortic. Res.* 6 1–15. 10.1038/s41438-019-0183-x 31666961 PMC6804945

[B33] Liu H. Ling Y. Gong Y. Sun Y. Hou L. Zhang B. (2007). DNA damage induces N-acetyltransferase NAT10 gene expression through transcriptional activation. *Mol. Cell Biochem.* 300 249–258. 10.1007/s11010-006-9390-5 17180247

[B34] Liu H. Ouyang B. Zhang J. Wang T. Li H. Zhang Y. (2012). Differential modulation of photosynthesis, signaling, and transcriptional regulation between tolerant and sensitive tomato genotypes under cold stress. *PLoS One* 7:e50785. 10.1371/journal.pone.0050785 23226384 PMC3511270

[B35] Lv W. T. Lin B. Zhang M. Hua X. J. (2011). Proline accumulation is inhibitory to *Arabidopsis* seedlings during heat stress. *Plant Physiol.* 156 1921–1933. 10.1104/pp.111.175810 21670222 PMC3149957

[B36] Ma X. Lv S. Zhang C. Yang C. (2013). Histone deacetylases and their functions in plants. *Plant Cell Rep.* 32 465–478. 10.1007/s00299-013-1393-6 23408190

[B37] Ma X. Yang C. Xia D. (2016). Characterization and expression analysis of histone deacetylases family RPD3/HDA1 in *Populus trichocarpa*. *Biol. Plant.* 60 235–243. 10.1007/s10535-015-0579-x

[B38] Mayfield S. P. Bennoun P. Rochaix J. (1987). Expression of the nuclear encoded OEE1 protein is required for oxygen evolution and stability of photosystem II particles in *Chlamydomonas reinhardtii*. *EMBO J.* 6 313–318. 10.1002/j.1460-2075.1987.tb04756.x 3556163 PMC553397

[B39] McFarlane C. R. Shah N. R. Kabasakal B. V. Echeverria B. Cotton C. A. Bubeck D. (2019). Structural basis of light-induced redox regulation in the Calvin-Benson cycle in cyanobacteria. *Proc. Natl. Acad. Sci. U. S. A.* 116 20984–20990. 10.1073/pnas.1906722116 31570616 PMC6800369

[B40] Min L. Li Y. Hu Q. Zhu L. Gao W. Wu Y. (2014). Sugar and auxin signaling pathways respond to high-temperature stress during anther development as revealed by transcript profiling analysis in cotton. *Plant Physiol.* 164 1293–1308. 10.1104/pp.113.232314 24481135 PMC3938621

[B41] Mo R. Yang M. Chen Z. Cheng Z. Yi X. Li C. (2015). Acetylome analysis reveals the involvement of lysine acetylation in photosynthesis and carbon metabolism in the model cyanobacterium Synechocystis sp. PCC 6803. *J. Proteome Res.* 14 1275–1286. 10.1021/pr501275a 25621733

[B42] Nallamilli B. R. Edelmann M. J. Zhong X. Tan F. Mujahid H. Zhang J. (2014). Global analysis of lysine acetylation suggests the involvement of protein acetylation in diverse biological processes in rice (*Oryza sativa*). *PLoS One* 9:e89283. 10.1371/journal.pone.0089283 24586658 PMC3930695

[B43] Nie Z. Zhu H. Zhou Y. Wu C. Liu Y. Sheng Q. (2015). Comprehensive profiling of lysine acetylation suggests the widespread function is regulated by protein acetylation in the silkworm, *Bombyx mori*. *Proteomics* 15 3253–3266. 10.1002/pmic.201500001 26046922

[B44] Ou L. Wei G. Zhang Z. Dai X. Zou X. (2015). Effects of low temperature and low irradiance on the physiological characteristics and related gene expression of different pepper species. *Photosynthetica* 53 85–94. 10.1007/s11099-015-0084-7

[B45] Pan J. Ye Z. Cheng Z. Peng X. Wen L. Zhao F. (2014). Systematic analysis of the lysine acetylome in *Vibrio parahemolyticus*. *J. Proteome Res.* 13 3294–3302. 10.1021/pr500133t 24874924

[B46] Pavangadkar K. Thomashow M. F. Triezenberg S. J. (2010). Histone dynamics and roles of histone acetyltransferases during cold-induced gene regulation in *Arabidopsis*. *Plant Mol. Biol.* 74 183–200. 10.1007/s11103-010-9665-9 20661629

[B47] Philp A. Rowland T. Perez-Schindler J. Schenk S. (2014). Understanding the acetylome: translating targeted proteomics into meaningful physiology. *Am. J. Physiol. Cell Physiol.* 307 C763–C773. 10.1152/ajpcell.00399.2013 25186010 PMC4216940

[B48] Pivonia S. Cock A. Levita R. Etiel E. Cohen R. (2012). Low temperatures enhance winter wilt of pepper plants caused by Pythium sp. *Phytoparasitica* 40 525–531. 10.1007/s12600-012-0254-0

[B49] Rojas-González J. A. Soto-Súarez M. García-Díaz Á Romero-Puertas M. C. Sandalio L. M. Mérida Á, et al. (2015). Disruption of both chloroplastic and cytosolic FBPase genes results in a dwarf phenotype and important starch and metabolite changes in *Arabidopsis thaliana*. *J. Exp. Bot.* 66 2673–2689. 10.1093/jxb/erv062 25743161 PMC4986871

[B50] Savitch L. V. Leonardos E. D. Krol M. Jansson S. Grodzinski B. Huner N. (2002). Two different strategies for light utilization in photosynthesis in relation to growth and cold acclimation. *Plant Cell Environ.* 25 761–771. 10.1046/j.1365-3040.2002.00861.x

[B51] Smith-Hammond C. L. Swatek K. N. Johnston M. L. Thelen J. J. Miernyk J. A. (2014). Initial description of the developing soybean seed protein Lys-Nε-acetylome. *J. Proteomics* 96 56–66. 10.1016/j.jprot.2013.10.038 24211405

[B52] Sridha S. Wu K. (2006). Identification of AtHD2C as a novel regulator of abscisic acid responses in *Arabidopsis*. *Plant J.* 46 124–133. 10.1111/j.1365-313X.2006.02678.x 16553900

[B53] To T. K. Nakaminami K. Kim J. M. Morosawa T. Ishida J. Tanaka M. (2011). *Arabidopsis* HDA6 is required for freezing tolerance. *Biochem. Biophys. Res. Commun.* 406 414–419. 10.1016/j.bbrc.2011.02.058 21329671

[B54] Vanselow C. Weber A. P. Krause K. Fromme P. (2009). Genetic analysis of the Photosystem I subunits from the red alga, *Galdieria sulphuraria*. *Biochim. Biophys. Acta* 1787 46–59. 10.1016/j.bbabio.2008.10.004 19007746

[B55] Walley J. W. Shen Z. McReynolds M. R. Schmelz E. A. Briggs S. P. (2018). Fungal-induced protein hyperacetylation in maize identified by acetylome profiling. *Proc. Natl. Acad. Sci. U. S. A.* 115 210–215. 10.1073/pnas.1717519115 29259121 PMC5776827

[B56] Wise R. R. (1995). Chilling-enhanced photooxidation: the production, action and study of reactive oxygen species produced during chilling in the light. *Photosynth. Res.* 45 79–97. 10.1007/BF00032579 24301474

[B57] Wu X. Oh M. H. Schwarz E. M. Larue C. T. Sivaguru M. Imai B. S. (2011). Lysine acetylation is a widespread protein modification for diverse proteins in *Arabidopsis*. *Plant Physiol.* 155 1769–1778. 10.1104/pp.110.165852 21311030 PMC3091122

[B58] Xing S. Poirier Y. (2012). The protein acetylome and the regulation of metabolism. *Trends Plant Sci.* 17 423–430. 10.1016/j.tplants.2012.03.008 22503580

[B59] Yang Q. S. Gao J. He W. D. Dou T. X. Ding L. J. Wu J. H. (2015). Comparative transcriptomics analysis reveals difference of key gene expression between banana and plantain in response to cold stress. *BMC Genomics* 16:446. 10.1186/s12864-015-1551-z 26059100 PMC4461995

[B60] Yang Y. J. Chang W. Huang W. Zhang S. B. Hu H. (2017). The effects of chilling-light stress on photosystems I and II in three Paphiopedilum species. *Bot. Stud.* 58 1–12. 10.1186/s40529-017-0208-4 29177684 PMC5702284

[B61] Zhang W. Zhang H. Ning L. Li B. Bao M. (2016). Quantitative proteomic analysis provides novel insights into cold stress responses in petunia seedlings. *Front. Plant Sci.* 7:136. 10.3389/fpls.2016.00136 26941746 PMC4766708

