# Supplementary figures and images for: Comprehensive Proteome and Lysine Acetylome Analysis Reveals the Widespread Involvement of Acetylation in Cold Resistance of Pepper (Capsicum annuum L.)
Source: Front Plant Sci. 2021 Aug 27;12:730489. doi: 10.3389/fpls.2021.730489 (PMC8429487; doi:10.3389/fpls.2021.730489)

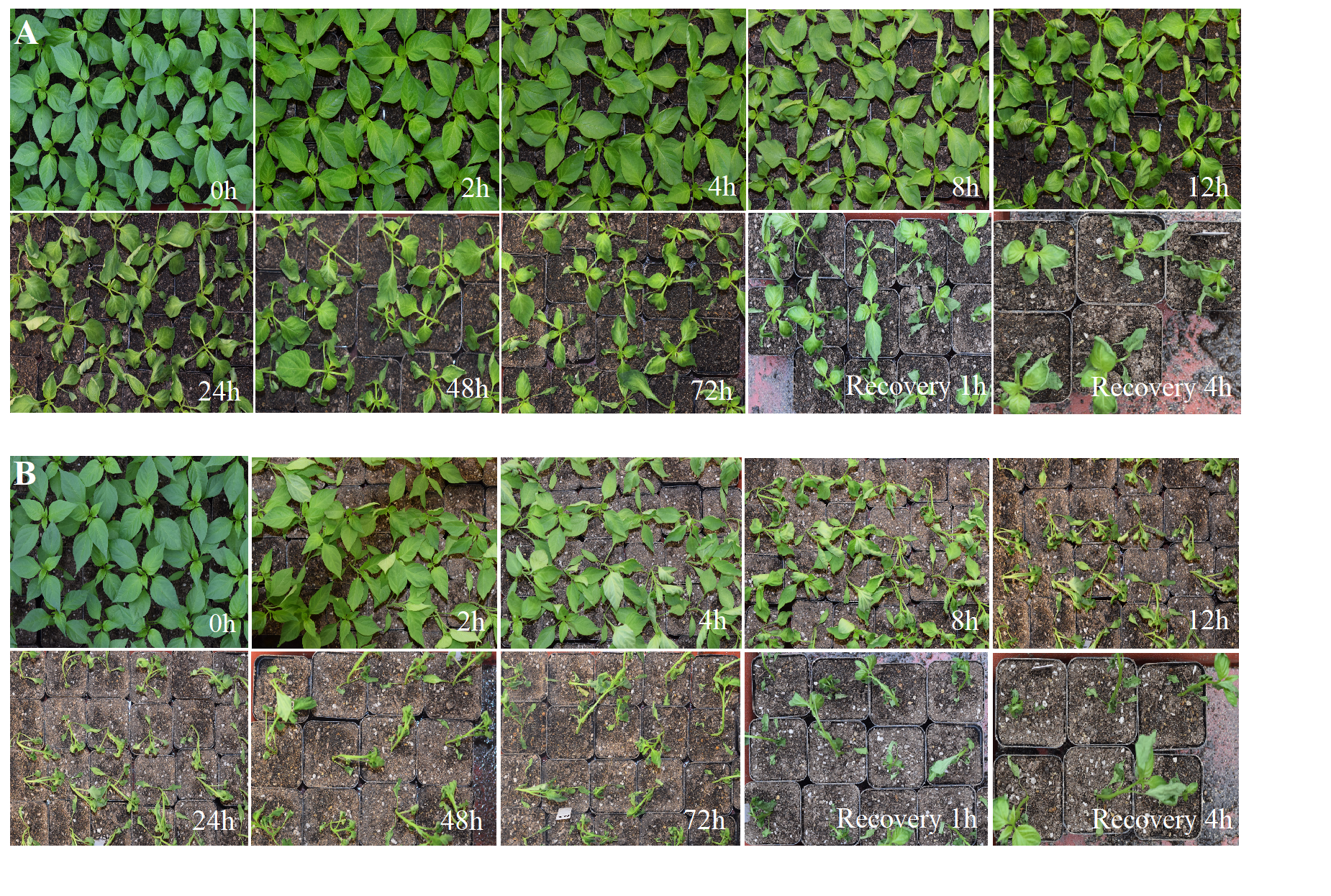

Supplement: Supplementary Figure 1 — Phenotypic changes in the two pepper varieties during cold stress and recovery. [file Image_1.TIF]

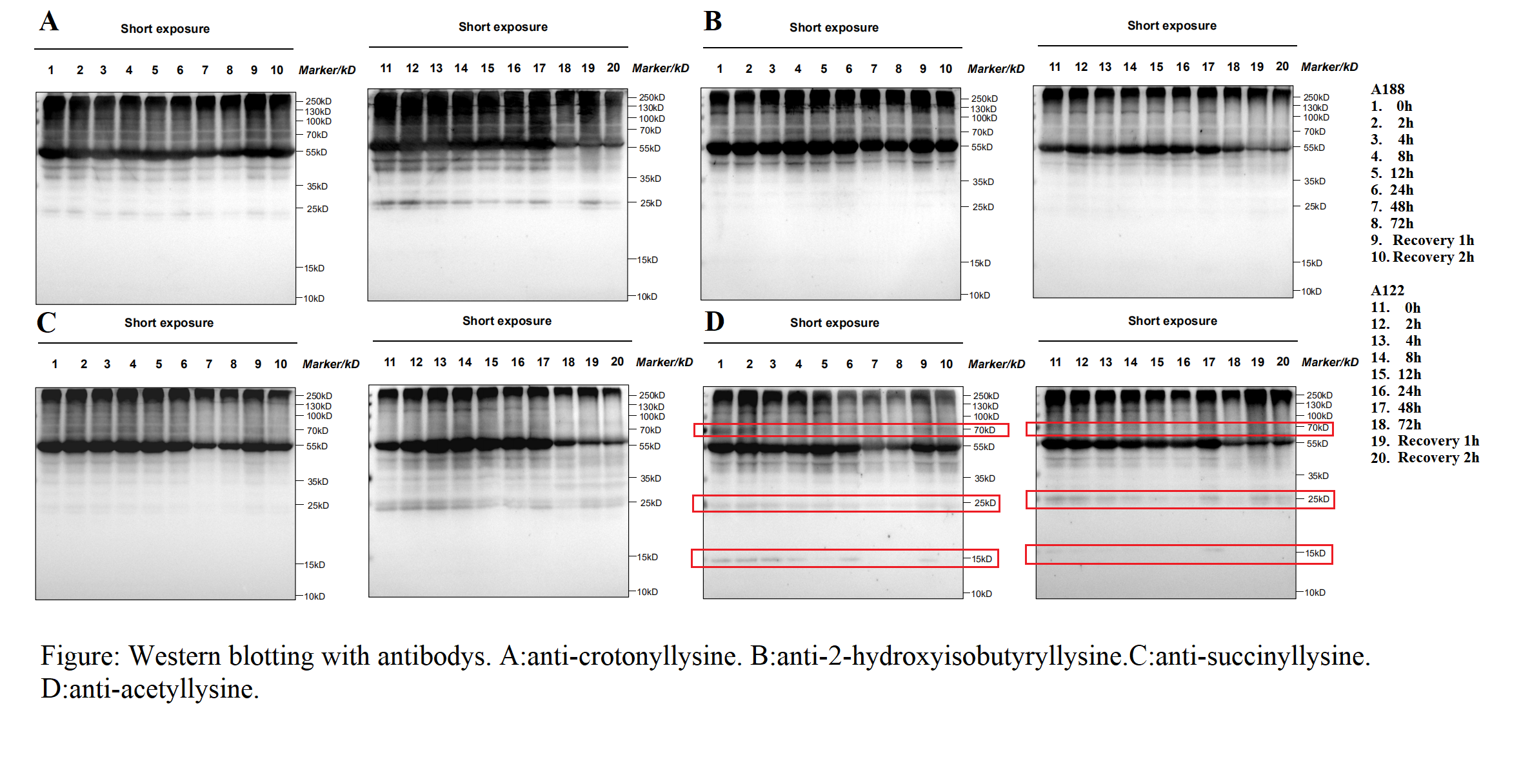

Supplement: Supplementary Figure 2 — Western blotting with antibodies. (A) Anti-crotonyllysine. (B) Anti-2-hydroxyisobutyryllysine. (C) Anti-succinyllysine. (D) Anti-acetyllysine. [file Image_2.TIF]

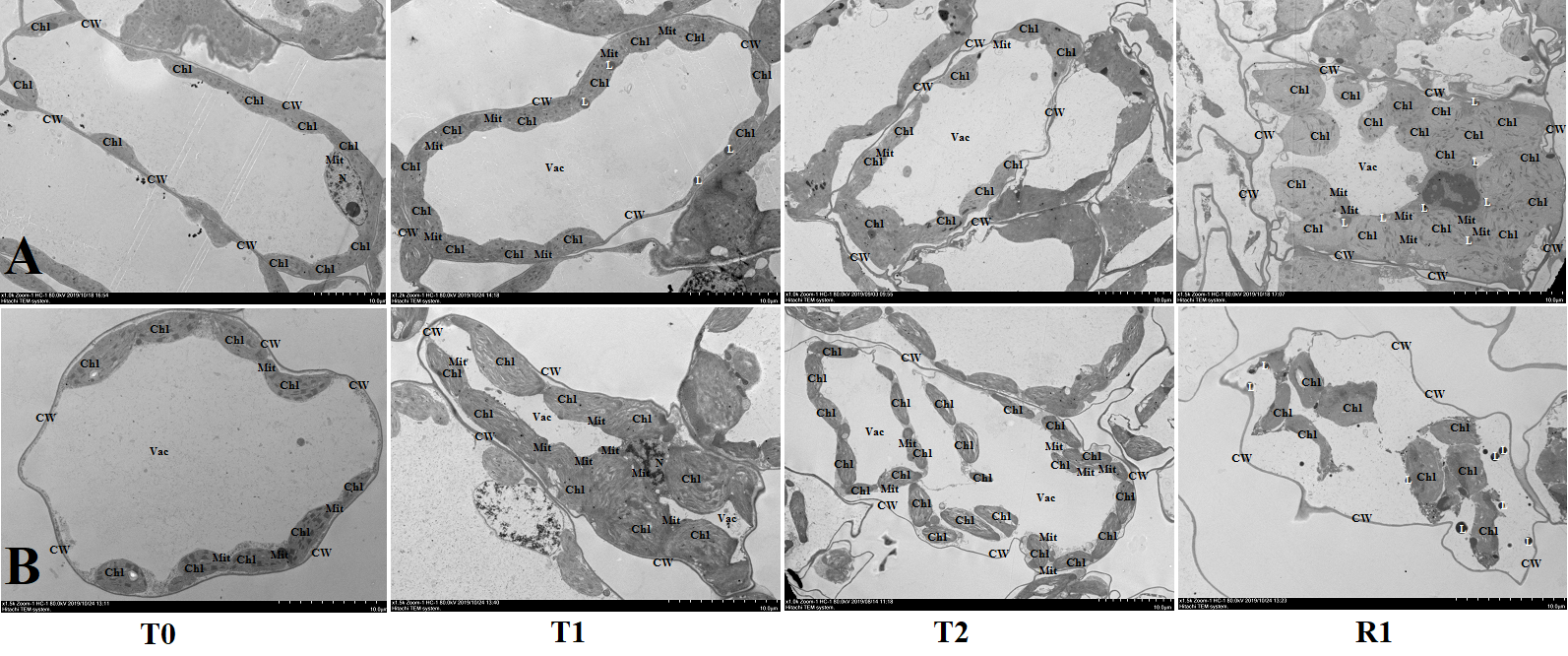

Supplement: Supplementary Figure 3 — Transmission electron microscope analysis of A188 (cold-resistant variety) and A122 (cold-sensitive variety) under cold stress and recovery stages. [file Image_3.TIF]

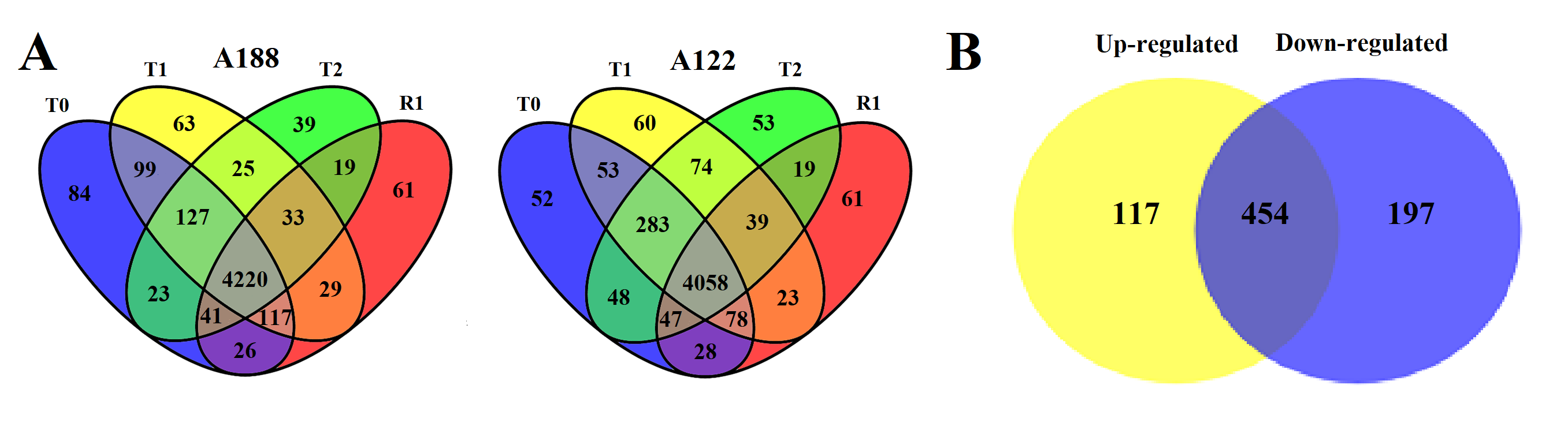

Supplement: Supplementary Figure 4 — Venn diagrams of samples. (A) Numbers of proteins identified in each treatment. (B) Numbers of differentially expressed acetylation proteins under cold stress. [file Image_4.TIF]

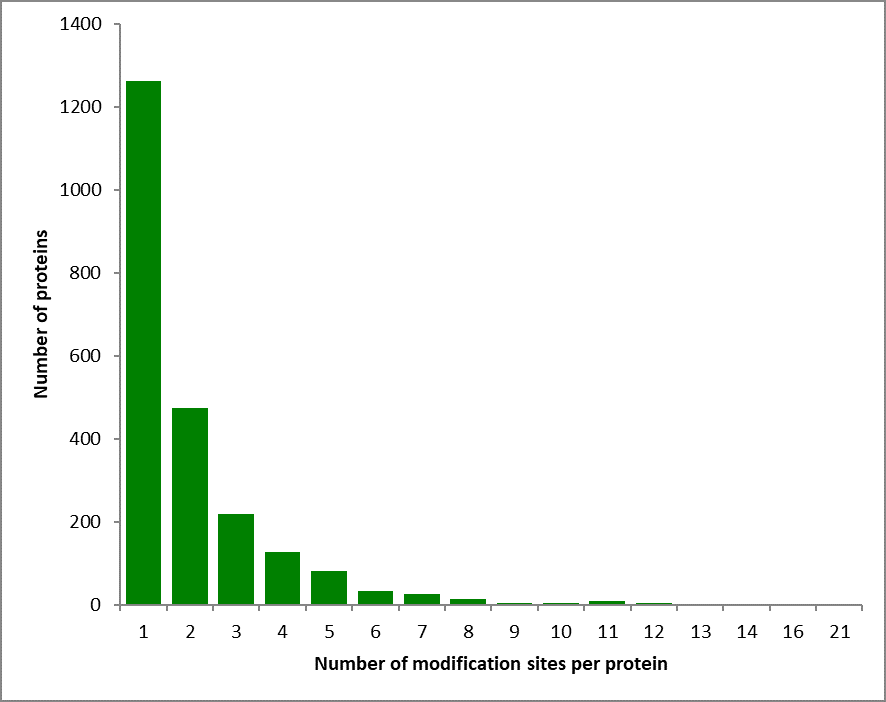

Supplement: Supplementary Figure 5 — Statistics on the number of acetylation sites in proteins. [file Image_5.TIF]

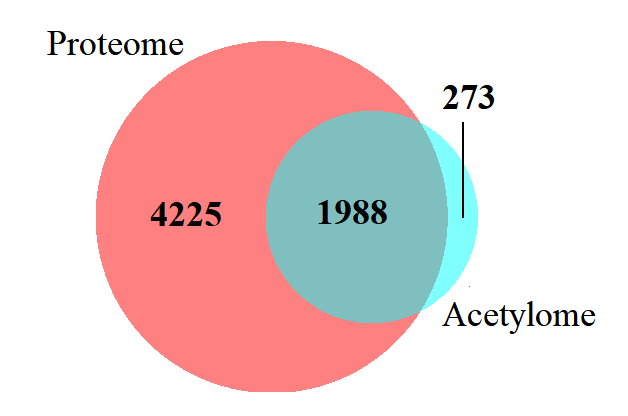

Supplement: Supplementary Figure 6 — Statistics of co-identified proteins in proteome and acetylome. [file Image_6.TIF]

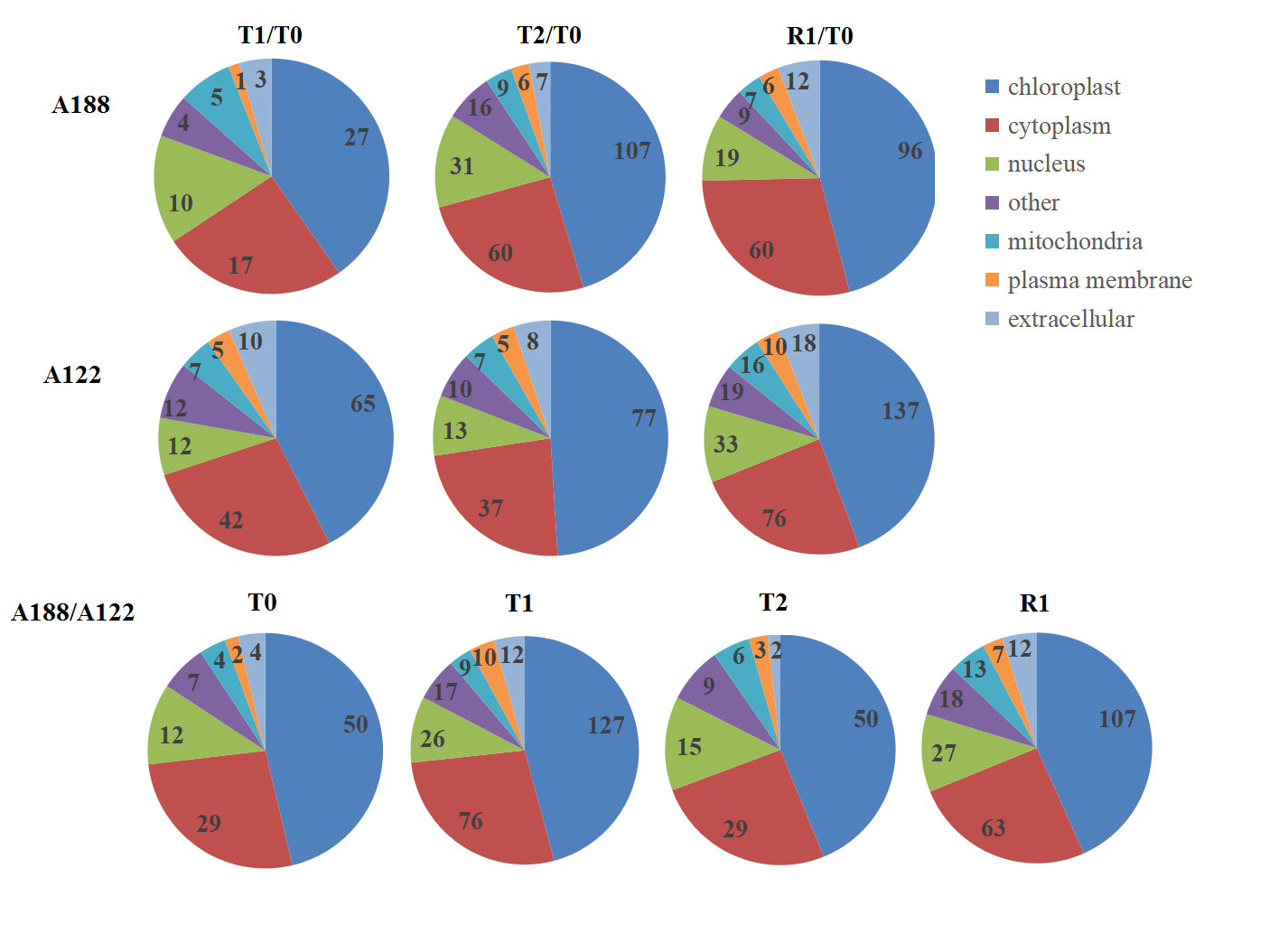

Supplement: Supplementary Figure 7 — Subcellular localization analysis of acetylated proteins. [file Image_7.TIF]
